# Micro-fragmented collagen hydrogel wound dressing: Enhanced porosity facilitates elevated stem cell survival and paracrine effects for accelerated wound maturation

**DOI:** 10.1016/j.mtbio.2025.101678

**Published:** 2025-03-22

**Authors:** Changgi Hong, Youngseop Lee, Haeun Chung, Dongwoo Kim, Jeongmin Kim, Jong-Wan Kim, Kangwon Lee, Sang-Heon Kim

**Affiliations:** a Center for Biomaterials, Biomedical Research Institute, Korea Institute of Science and Technology (KIST), 02792, Seoul, Republic of Korea; b Department of Applied Bioengineering, Graduate School of Convergence Science and Technology, Seoul National University, Seoul, 08826, Republic of Korea; c Division of Bio-Medical Science and Technology, KIST School, Korea University of Science and Technology, Seoul, 02792, Republic of Korea; d Research Institute for Convergence Science, Seoul National University, Seoul, 08826, Republic of Korea; e S.Biomedics Co., Ltd., Seoul, 04797, Republic of Korea

**Keywords:** Wound healing, Micro-fragmentation, Hydrogel wound dressing, hADSCs, Stem cell therapy

## Abstract

Human Adipose-derived stem cells (hADSCs), known for their mesenchymal stem cell properties, including multilineage differentiation and self-renewal, hold significant promise for chronic wound regeneration. Typically, hADSCs are utilized in cellular aggregates or hydrogels to enhance therapeutic efficacy. However, limitations such as reduced cell viability, inadequate mass transfer rates, and diminished paracrine effects hinder their clinical applications. This study explores an innovative approach by encapsulating hADSCs within a collagen/hyaluronic acid micro-fragmented collagen hydrogel wound dressing (MCWD). The resulting micro-fragmented collagen hydrogel-hADSC composite created through the integration of micro-sized hydrogel units and cells demonstrated markedly improved cell viability and activity, as well as superior therapeutic outcomes compared to conventional cell aggregates (CA) and collagen hydrogel wound dressings (CWD*). In vitro* assessments showed that the highly porous structure of MCWD promotes better mass transfer and enhances the viability and cytokine production of hADSCs associated with the paracrine effect. *In vivo* experiments further validated the effectiveness of the MCWD, revealing significant enhancements in cell proliferation, skin thickness restoration, collagen maturation, and blood vessel formation. These findings underscore the potential of MCWD as an advanced solution for wound healing applications.

## Highlights

• The high-porosity architecture of MCWD significantly enhances stem cell viability and functional activity.
• MCWD promotes the activation of skin-regenerating cells *in vitro*, which translates to improved skin regeneration and accelerated tissue maturation *in vivo.*
• Introduces a high-performance hydrogel platform engineered for stem cell-based therapies in chronic wound management.

## Graphical abstract

**Figure:**
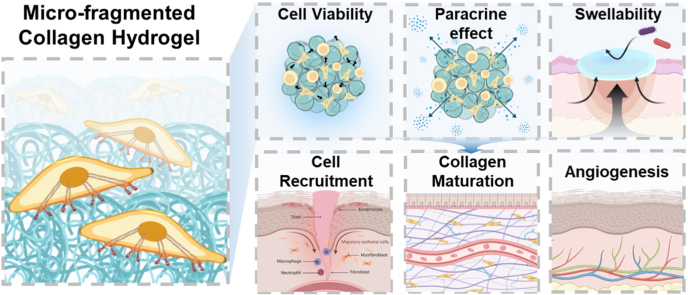

## 1 Introduction

Stem cell therapy has emerged as a promising strategy for wound healing, offering potential benefits such as enhanced neovascularization, accelerated wound closure, reduced scar formation, and improved collagen maturation [1,2]. Among the various types of stem cells, human adipose-derived stem cells (hADSCs) have gained considerable attention due to their ability to support skin regeneration. This is achieved through the secretion of angiogenic and cell survival factors, which operate in a paracrine manner to promote vascularization and improve the survival of cells involved in wound repair [3,4]. Despite these advantages, the long-term efficacy of stem cell therapy has been limited by the short retention time of stem cells at the wound site [5,6]. Challenges such as cell lysis, low nutrients, anoikis, oxidative stress, and immune response can rapidly reduce the number of transplanted cells, thereby compromising therapeutic outcomes.

To address these issues, researchers have developed technologies, including cell aggregates and hydrogels, aimed at enhancing the effectiveness and functionality of transplanted stem cells [7,8]. These technologies are designed to prolong the paracrine effects of hADSCs at wound sites, ensuring adequate delivery, survival, integration, proliferation, and activation of the cells [9,10]. For instance, studies have shown that cell aggregates exhibit improved tolerance to reactive oxygen species (ROS), enhanced cell viability, and greater angiogenic properties, leading to more effective wound healing in mouse models [11]. Similarly, hydrogels encapsulating hADSCs have demonstrated promising regenerative capabilities by accelerating wound healing through reduced inflammation, increased angiogenesis, and enhanced re-epithelialization [12]. However, both cell aggregates and hydrogels have limitations in maintaining prolonged paracrine factor secretion and cell viability over extended periods [5,13]. Cell aggregates, while effective in secreting paracrine factors necessary for tissue regeneration, often suffer from issues related to survival, extracellular matrix interaction, and mass transfer, resulting in necrotic core formation. Hydrogels, though they can mimic the extracellular matrix, have a low surface-to-volume ratio that impairs mass transfer and scalability for wound dressing applications, affecting cell viability and cytokine release. These limitations highlight the need for further research and innovative strategies to optimize stem cell therapy approaches.

Recently, the design of micro-scale scaffolds has emerged as a promising biomaterial technique for tissue engineering and stem cell transplantation [14]. Microgels, which are macroscopic aggregates created by assembling small hydrogel fragments with cells, offer a high surface-to-volume ratio and short diffusion distances. This design facilitates enhanced nutrient, oxygen, and bioactive factor diffusion to encapsulated stem cells, thereby promoting their viability and activity [15]. Research has demonstrated that the interconnected pore network of microgels supports efficient exchange of metabolic waste products, mitigates necrotic core formation, and maintains a favorable microenvironment for sustained stem cell activation [16]. Despite the promise of microgels, their manufacturing process has traditionally relied on inefficient bottom-up approaches due to the self-assembly of collagen hydrogels, which increases yield stress and makes it challenging to fragment the material into small particles using top-down methods. Techniques like lithography, microfluidic emulsions, and electro-hydrodynamic spraying have been explored but face scalability issues for large-scale applications [17]. To overcome these challenges, a top-down manufacturing approach for collagen-based micro-fragmented collagen hydrogel has been developed, utilizing atelocollagen to prevent self-assembly by removing telopeptides from collagen filaments and adding hyaluronic acid to form polyion complex that further inhibits self-assembly [[18], [19], [20]]. These methods effectively reduced collagen fibrillogenesis, allowing for the mechanical fragmentation of the collagen hydrogel into micro-scale hydrogel approximately 40 μm in length and 20 μm in width, which can be crosslinked during cellular interactions [15].

**Fig. 1:**
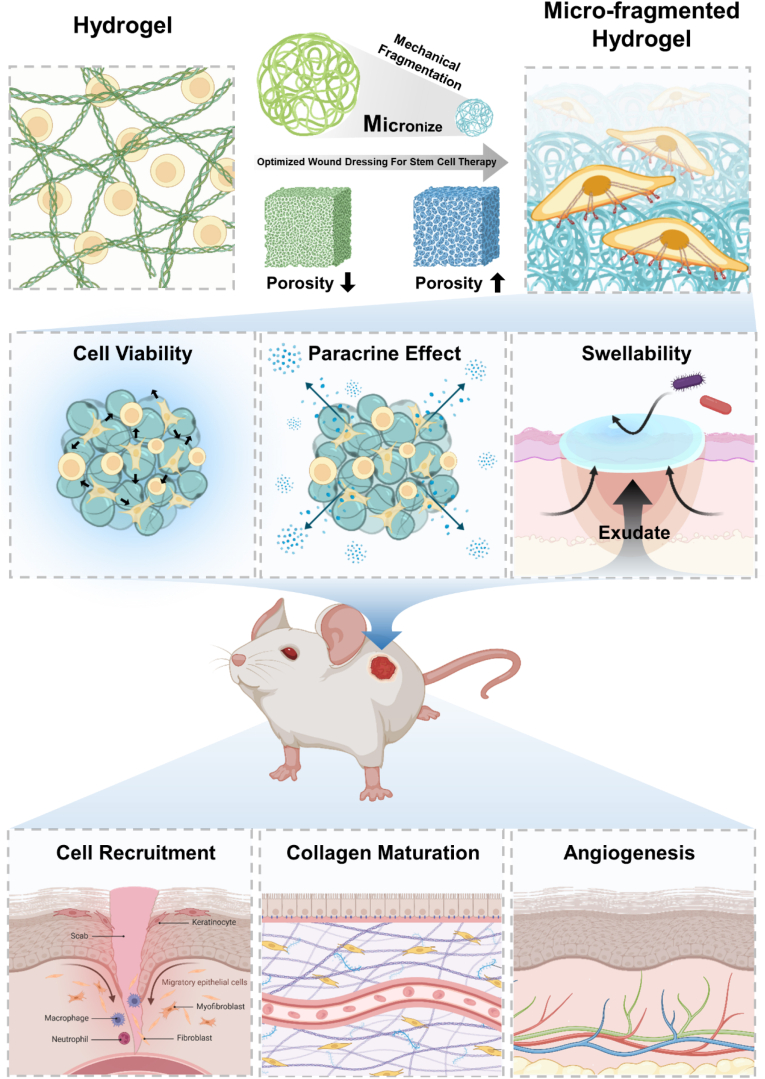
Schematic illustration of the use of collagen/hyaluronic acid micro-fragmented collagen hydrogel-based stem cell wound dressing (MCWD) therapy for wound healing. Figures were created using the online platform Biorender.com.

This study evaluated the potential of collagen micro-fragmented collagen hydrogels as cell-assembling scaffolds for wound dressings, comparing their performance with conventional collagen hydrogels. Stochastic Optical Reconstruction Microscopy imaging (STORM), scanning electron microscopy (SEM), and micro-computed Tomography (μCT) were utilized to analyze the porosity and structure of the cell-scaffold assemblies. Viability assays, enzyme-linked immunosorbent assay (ELISA), *in vitro* wound healing, and tube formation assays were performed to assess the biological properties of micro-fragmented collagen hydrogel-hADSCs assemblies for wound dressing applications. Additionally, the mechanical properties of the MCWD were evaluated to ensure their suitability as a wound dressing. Finally, *in vivo* full-thickness wound models were employed to evaluate key outcomes, including cell proliferation, collagen maturation, and angiogenesis. The results demonstrated that the micro-fragmented collagen hydrogel platform offers significant advantages as a biomaterial for wound dressings in stem cell therapy. This approach presents a promising solution for improving therapeutic outcomes in chronic wound management and suggests potential for broader application in the field of wound dressing (Fig. 1).

## 2 Materials and methods

### 2.1 Fabrication of fragmented Collagen/HA solution

The micro-fragmented collagen hydrogel preparation was conducted following the method described in our previous publication [15]. Briefly, stock solutions of atelocollagen (25 % w/w, MSBio, Inc., Seoul, South Korea) and sodium hyaluronate (HA) (10 % w/w, Contipro Inc., Dolni Dorouc, Czech Republic) were prepared separately. Atelocollagen was dissolved in 0.01 N hydrochloric acid, while HA was dissolved in phosphate-buffered saline (PBS). The atelocollagen and HA solutions were mixed in a 4:5 vol ratio and carefully homogenized on ice. The pH of the mixture was adjusted to 7.1–7.3 using 2 N NaOH or 1 N HCl. PBS was added to reach the desired final volume, and the mixture was incubated at 37 °C for 1 h to promote collagen self-assembly. Subsequently, the collagen/HA gel was transferred to a new tube containing CEFOgro media (CEFO Co., Seoul, South Korea) and glass beads. The solution was agitated at 1500 revolutions per minute (rpm) for 30 s using a Mini-Beadbeater-24 (BioSpec, Bartlesville, OK, USA). The resultant fragmented collagen solution was then filtered using a 100-μm filter under vacuum and stored at 4 °C. Notably, the micro-fragmented collagen hydrogel remains in a suspension state and does not undergo hydrogel formation, even under conditions that promote fibrillogenesis, such as incubation at 37 °C. Hydrogel assembly is triggered exclusively through interaction with cells during incubation, underscoring its potential as a dynamic and cell-responsive biomaterial tailored for advanced tissue engineering applications. Due to these intrinsic material properties, the micro-fragmented collagen hydrogel alone cannot be used as an independent experimental group, as it lacks structural integrity.

### 2.2 Preparation of CA, CWD, and CWMD

Human adipose-derived stem cells (hADSCs) were obtained from S. Biomedics (Seoul, South Korea) and authenticated according to the guidelines established by the International Society for Cell Therapy. The cells were cultured under standard conditions at 37 °C with 5 % CO_2_ in a humidified incubator. Passage 5 hADSCs (5 × 10^5^ cells) were utilized for all experiments and incorporated into cell aggregates (CA), collagen wound dressing (CWD), and micro-fragmented collagen hydrogel wound dressing (MCWD) (Fig. S1). CA was prepared by suspending hADSCs in 50 μL of CEFOgro medium, which was then dispensed onto a sterilized ultra-low attachment 24-well plate lid (Corning® Costar®, NY, USA). The lid was gently inverted over wells containing a small volume of PBS to prevent evaporation and incubated at 37 °C with 5 % CO_2_ for 6 h. The cells within the droplet migrated downward due to gravitational forces, promoting cell-cell interactions [21]. After incubation, the cell aggregates were transferred to 2 mL of CEFOgro media in an ultra-low attachment 24-well plate. CWD was prepared by neutralizing a 0.5 % atelocollagen solution that had been diluted in 0.01 N HCl using 1 N NaOH. Subsequently, 750 μL of the neutralized solution was aliquoted into an ultra-low attachment 24-well plate. The hADSCs were suspended in 500 μL of CEFOgro medium and mixed with the neutralized collagen solution in the 24-well plate. Additional CEFOgro medium was added to achieve a final volume of 2 mL. The plate was incubated at 37 °C with 5 % CO_2_ for 24 h, allowing the collagen solution to undergo fibrillogenesis and form CWD. The 0.5 % collagen concentration was selected to optimize the mechanical properties for wound dressing functionality while minimizing excessive fibrillogenesis-induced shrinkage, thus ensuring structural integrity and the viability of the encapsulated hADSCs [[22], [23], [24]]. MCWD was prepared by homogenizing 750 μL of fragmented collagen/hyaluronic acid solution with hADSCs suspended in 500 μL of CEFOgro medium in an ultra-low attachment 24-well plate. After thorough mixing, CEFOgro medium was added to achieve a final volume of 2 mL. The 24-well plate was incubated at 37 °C with 5 % CO_2_ for 24 h, allowing the cells to interact and form MCWD undisturbed. MCWD was achieved through the interaction between the mechanically fragmented collagen/hyaluronic acid and hADSCs. Upon adhesion, the contraction of actin filaments within the cells facilitated the aggregation of collagen hydrogel fragments, culminating in the formation of a cohesive structure [15]. Throughout the experiment, the medium was replaced daily with fresh CEFOgro medium to maintain optimal cell culture conditions for all sample groups.

### 2.3 Super-resolution fluorescence microscopy

The thickness of collagen fibers in CWD and MCWD was assessed using 3D stochastic optical reconstruction microscopy (STORM) imaging. Samples were first fixed in 4 % paraformaldehyde at 37 °C for 2 h, followed by washing with distilled water. The samples were then immersed in a 25 % (w/w) optimal cutting temperature (OCT) solution for 24 h, transferred to a 100 % (w/w) OCT solution for an additional 24 h, and rapidly frozen in liquid nitrogen. Frozen samples were cryo-sectioned into 5-μm-thick slices and mounted on glass slides. Mounted samples were rinsed with 70 % and 100 % ethanol, then blocked with a 5 % bovine serum albumin (BSA) solution for 2 h to minimize non-specific binding. Collagen fibers were labeled with Alexa Fluor 647 (Invitrogen, Waltham, MA, USA) for 2 h, after which the samples were rinsed with PBS to remove unbound dye. The samples were placed on 1.5H coverslips and mounted with a STORM imaging buffer containing 100 mM cysteamine, 10 % glucose, 0.8 mg/mL glucose oxidase, 40 μg/mL catalase, 50 mM Tris-HCl, and 10 mM NaCl in PBS at pH 8.0. The samples were sealed with nail polish prior to imaging. Imaging was performed using a custom STORM system equipped with a 100 × /1.45 NA objective lens. This system captures the astigmatic point spread functions of Alexa Fluor 647 and CF568 dye molecules to determine their 3D positions. Single-molecule movies of collagen fibers were recorded at a frame rate of 50 frames per second, encompassing a total of 20,000 frames. Excitation was provided by a 642 nm and 542 nm laser at an average illumination intensity of approximately 10 kW/cm^2^, with a low-power 405 nm laser used for activation. The STORM video data were localized to reconstruct 3D STORM images, following the procedures outlined in a previous study [25]. The lateral and axial cross-sectional intensity profiles of 5 random fibers from the reconstructed images were analyzed to compute fiber thickness. For each fiber, the full width at half-maximum intensity was measured in both the lateral and axial dimensions, and the fiber thickness was calculated as √ (axial^2^ + lateral^2^) (n = 10). Colocalization analysis of two-color STORM images was performed using the JACoP plugin in ImageJ [26]. Automatic thresholding was applied using the Costes regression approach to optimize the threshold levels for each color channel. Following thresholding, Manders' coefficient, overlap coefficient, and Pearson's correlation coefficient were calculated to quantitatively assess the degree of colocalization between β-actin and collagen (n = 5).

### 2.4 Scanning electron microscopy (SEM)

For SEM imaging, samples were fixed in a 4 % paraformaldehyde solution for 2 h. Following fixation, the samples were dehydrated through a graded ethanol series (70 %, 80 %, 90 %, and 100 %). Dehydrated samples were desiccated using a freeze dryer (Ilshin Bio Base, Gyeonggi, South Korea) for 48 h. The desiccated samples were then coated with platinum using a sputter coater (SPI-module sputter coater; SPI Supplies, West Chester, PA, USA). Coated samples were examined using a scanning electron microscope (Teneo Volume Scope, FEI; Hillsboro, OR, USA) at an acceleration voltage of 15 kV [27].

### 2.5 Micro-computed tomography (μCT)

μCT imaging was utilized to evaluate the porosity of the 3D structures of CWD and MCWD [15]. Samples were fixed in a 4 % paraformaldehyde solution and stained with 5 % phosphotungstic acid (Tokyo Chemical Industry, Tokyo, Japan) in 70 % ethanol for 3 days. Following staining, samples were placed in PBS. Computerized tomography scans were performed using a μCT machine (Skyscan 1172, Bruker, Billerica, MA, USA) with the following parameters: an aluminum filter thickness of 0.5 mm, an X-ray tube voltage of 45 kV, a tube current of 134 μA, a pixel size of 3 μm, and a scanning angle range of 360°. The acquired data were reconstructed using NRecon software and analyzed with CTAn software (Bruker) to determine the porosity of the samples (n = 3).

### 2.6 Swelling ratio

The swelling capacity of the CWD and MCWD was evaluated by measuring their weight changes at 37 °C over different time intervals [28]. Prior to the swelling test, the samples were lyophilized and subsequently immersed in PBS to allow swelling. Excess PBS on the sample surfaces was carefully removed using filter paper before each weight measurement, and the samples were weighed accurately (n = 3). The swelling ratio was calculated using the following formula:

$$\text{Swelling}\;\text{Ratio}:\left(W_{t}-W_{0}\right)/W_{0}\;x\;100$$

where.

W_t_: Weight of the swollen sample at time t
W_0_: Initial weight of the lyophilized sample.

### *2.7* *In vitro* biodegradation assay

To assess the rate of enzymatic biodegradation *in vitro*, CWD and MCWD samples were lyophilized [29]. Subsequently, 10 units of collagenase (Gibco, Thermo Fisher Scientific, Waltham, MA, USA) were dissolved in 1 mL of PBS and incubated with the samples at 37 °C (n = 3). The biodegradation ratio was calculated using the formula:

$$\text{Biodegradation}\;\text{Ratio}:W_{t}\;/\;W_{0}\;x\;100$$

where.

W_t_: Weight of the sample at time t
W_0_: Initial weight of the lyophilized sample

### 2.8 Rheological and mechanical evaluation

The mechanical properties of CWD and MCWD were assessed by measuring their viscoelastic behavior. The storage modulus (G′) and loss modulus (G″) were determined using an MCR 102 rheometer (Anton-Paar, Graz, Austria) to evaluate the elasticity and viscosity of the samples. Dynamic strain sweeps were conducted at a frequency of 1 Hz and a temperature of 25 °C until a plateau was reached [30]. Measurements were performed on days 3, 7, and 14 to assess changes in the mechanical properties of the constructs over time.

### 2.9 Evaluation of cell viability

Cell viability was assessed by quantifying ATP content using the CellTiter-Glo® 3D Cell Viability Assay Kit (Promega, Madison, WI, USA) following the manufacturer protocol. Luminescence was measured using a GloMax® Discover System (Promega, Madison, WI, USA) (n = 3). For cell quantification, the fluorometric PicoGreen double-stranded DNA assay was performed following the instructions provided by the manufacturer (Quant-iT™ PicoGreen® dsDNA kit, P7589, Invitrogen). Briefly, samples collected after each time point were rinsed with PBS, placed in microtubes containing 500 μL of PBS, and homogenized by sonication. The homogenized samples were then diluted with an equal volume of TE buffer, and 100 μL aliquots of the diluted solution were added to a 96-well plate, followed by the addition of 100 μL of PicoGreen reagent (200x). The mixture was incubated for 10 min at 25 °C, and fluorescence was measured at 480 nm (excitation) and 520 nm (emission) using a GloMax Discover Multimode Microplate Reader (Promega). DNA concentration was calculated using a standard curve (0–2 μg/mL) correlating dsDNA quantity to fluorescence intensity (n = 3). To perform the Live/Dead cell viability assay, samples were stained with 20 μM calcein AM and 10 μM propidium iodide (Invitrogen) and incubated for overnight. Following incubation, samples were fixed with 4 % paraformaldehyde, embedded in OCT compound, and cryo-sectioned into 6-μm-thick slices. The sections were then mounted with a cover slip. Fluorescence images were captured using an LSM 700 confocal microscope (Zeiss, Oberkochen, Germany).

### 2.10 Enzyme-linked immunosorbent assay (ELISA)

To quantify the paracrine factors present within and secreted by CA, CWD, and MCWD, samples were cultured in 24-well plates with 2 mL of CEFOgro medium for three days. Following the culture period, samples were transferred to a 1M Tris-HCl solution (pH 7.2) (Biosesang, Gyeonggi, South Korea) and homogenized via sonication in an ice bath. Supernatants from the CA, CWD, and MCWD cultures in the 24-well plates were collected in accordance with the supplier instructions for further analysis. The concentrations of FGF2, VEGF, TGF-β1, HGF, IL-1β, and TIMP1 in the samples and supernatants were quantified using ELISA kits (R&D Systems, Minneapolis, MN, USA). Optical density was measured at 450 nm using a GloMax Discover Microplate Reader (Promega, Madison, WI, USA) (n = 3).

### 2.11 Dextran release assay

Fluorescein isothiocyanate (FITC)-dextran (Sigma-Aldrich, St. Louis, MO, USA) with molecular weights of 4, 10, and 40 kDa (a representative substance for small molecules) was introduced into both CWD and MCWD to examine cargo release efficiency. A total of 300 μg of FITC-dextran was included in CEFOgro medium during the synthesis of CWD and MCWD. Loading efficiency was determined by subtracting the quantity of dextran remaining in the medium after 24 h from the initial loading of 300 μg. Fluorescence in the medium was assessed using a GloMax Discover Multimode Microplate Reader (Promega) and quantified by comparing it to a dextran standard curve. To evaluate the cumulative release efficacy, samples were rinsed with PBS to remove any residual dextran on the surface and subsequently placed in a 2 mL of PBS. At each time point, 100 μL of PBS was collected for fluorescence intensity measurement, and an equal volume of fresh PBS was added back to the solution. Cumulative release was determined as the sum of dextran released at each time point (n = 3) [31].

### *2.12* *In vitro* wound healing assay

Human fibroblasts (5 × 10^4^ cells) (CEFO Co., Seoul, South Korea) were seeded into 24-well plates and allowed to proliferate for 48 h to facilitate cell adhesion and the formation of a confluent monolayer. The confluent monolayers were then scratched with a sterile pipette tip to create a wound approximately 400–500 μm wide. The culture medium and detached cells were promptly removed, and the wells were replaced with serum-free medium (SFM). CA, CWD, and MCWD were individually placed in transwell inserts, which were subsequently introduced into the 24-well plates containing SFM [29]. Fibroblast migration into the scratched area was monitored at baseline and after 24 h using an Axio Vert A1 phase contrast microscope (Zeiss). The wound gap area was measured at both time points using ImageJ software with the MRI Wound Healing Tool (National Institutes of Health, Maryland, USA) (n = 3).

### *2.13* *In vitro* HUVEC tube formation assay

Green fluorescent protein-tagged human umbilical vein endothelial cells (GFP-HUVECs) (Angio-Proteomie, MA) were cultured in endothelial growth medium (EGM-2, Lonza, Basel, Switzerland) supplemented with growth factors and antibiotics until passage four. For the tube formation assay, 24-well transwell culture plates (SPL Life Sciences, Pocheon, South Korea) were coated with growth factor-reduced basement membrane extract (Matrigel®, Corning) and incubated at 37 °C for 40 min. Serum-starved GFP-HUVECs were seeded at a density of 1 × 10^5^ cells per well in the lower compartment of the transwell plates and incubated at 37 °C for an additional 40 min. The upper compartments were loaded with different media conditions: (1) Endothelial cell growth basal medium (EBM) without supplements, (2) Endothelial growth medium (EGM) supplemented with angiogenic growth factors, (3) CA, (4) CWD, and (5) MCWD. Co-culture with GFP-HUVECs was maintained for 16 h. All experimental groups, except for the EGM, were cultured in EBM [15]. Tube formation in the transwells was visualized using a phase-contrast confocal microscope (Zeiss). The total number of node points in HUVEC tubes was quantified using ImageJ software (n = 3) [32].

### 2.14 CCK-8 cytotoxicity assay

Human fibroblasts (CEFO Co.) were seeded at a density of 5 × 10^4^ cells per well in 24-well plates and incubated for 24 h. Following media removal, the cells were treated with samples loaded in transwells containing SFM for 24 h in an incubator. Cell viability was assessed using the Cell Counting Kit-8 (CCK-8, Dojindo, Kumamoto, Japan) assay. Briefly, CCK-8 solution was added to each well and incubated for 2 h. Absorbance was measured at 450 nm using a GloMax Discover Multimode Microplate Reader (Promega) (n = 3).

### 2.15 Animal study: 8 mm full thickness skin wound splint model

The animal study was approved by the Korea Institute of Science and Technology (KIST-IACUC-2023-053-1) and conducted in accordance with the International Guide for the Care and Use of Laboratory Animals. CrlOri CD1 (ICR) mice (male, 6 weeks old) (Orient Bio, Seongnam, South Korea) were randomly assigned to four groups: control (no treatment), CA, CWD, and MCWD (n = 4). Prior to surgery, the mice were anesthetized via inhalation of isoflurane (Hana Pharm, Seoul, South Korea) in oxygen, and their dorsal skin was shaved and disinfected with alcohol. Full-thickness skin wounds (two wounds per mouse) were created using an 8-mm biopsy punch (Miltex, York, PA, USA) under sterile surgical conditions. A silicone splint was securely affixed to the skin around the incision to prevent localized skin contraction, facilitating healing by re-epithelialization and tissue regeneration. Each treatment sample was applied to the wound area, which was then covered with a Tegaderm™ film and Coban bandages (3M, St. Paul, Minnesota, USA) to prevent sample detachment. The Tegaderm™ film and Coban bandages were replaced every three days. Wound closure was evaluated on days 7 and 14 using digital images of the wounds. The wound area at each time point was quantitatively measured using ImageJ software and normalized to the initial wound area on day 0 to calculate the percentage of wound closure.

### 2.16 Histology and immunofluorescence staining

Following euthanasia by CO_2_ inhalation on days 7 and 14, skin wound tissues were excised, fixed in 10 % formalin, and embedded in paraffin blocks using the Excelsior AS tissue processor (Thermo Scientific, Waltham, MA, USA). The tissues were sectioned into 4-μm-thick slices spanning the entire wound area. Hematoxylin and eosin (H&E), Herovici (Abcam, Cambridge, UK), and immunofluorescence staining (CD-31, VEGF, and Ki-67) were performed according to previously established protocols [15]. Quantitative analysis of stained sections was conducted using ImageJ software, with measurements obtained from five random images per sample.

### *2.17* *In vivo* RT-qPCR

Gene expression patterns in wounds treated with CA, CWD, and MCWD were evaluated on days 3, 7, and 14 using RT-qPCR. Total mRNA was extracted using the TRIzol reagent (Invitrogen, Waltham, MA, USA) in accordance with the manufacturer instructions. RT-qPCR was performed in real-time with SYBR Premix Ex Taq (Takara, Shiga, Japan) on an ABI 7500 Real-Time System (Applied Biosystems, Waltham, MA, USA). Gene expression levels were normalized to glyceraldehyde 3-phosphate dehydrogenase (GAPDH), which served as the housekeeping gene. Primers were purchased from Bioneer (Daejeon, South Korea), and the sequences of the mouse primers used are provided in the supplementary materials (Fig. S2). Quantitative analysis was performed using the 2^-ΔΔCT^ method to measure the levels of collagen type I, collagen type III, VEGF, FGF2, TGF-β1, and GAPDH during the wound healing process (n = 3).

### 2.18 Immunofluorescence staining

For immunofluorescence staining of the 3D constructs, samples were prepared as frozen blocks in OCT and sectioned to a thickness of 6 μm. For *in vivo* wound tissues, samples were prepared as paraffin blocks and sectioned to a thickness of 4 μm. The sections were blocked with a solution containing 3 % BSA and 0.1 % Triton X-100 in PBS for 1 h. Following blocking, the sections were incubated overnight at 4 °C with primary antibodies diluted in the blocking solution. Subsequently, the sections were incubated with fluorescence-conjugated secondary antibodies, also diluted in the blocking solution, for 1 h at 25 °C. The samples were then mounted using Vectashield antifade mounting medium containing DAPI (Vector Laboratories Inc., Newark, CA, USA). Fluorescent images were acquired using a Zeiss LSM 700 confocal microscope (Zeiss). Detailed information on the antibodies used is provided in the supplementary materials (Fig. S3).

### 2.19 Statistical analysis

All statistical analyses were performed using PRISM 10 software (GraphPad, San Diego, CA, USA). Comparisons between multiple experimental groups were conducted using multiple t-tests or two-way ANOVA, assuming a Gaussian distribution and equal standard deviation (SD). Multiple comparison tests were performed with a confidence level of 95 %. Statistical significance is indicated as ∗p < 0.05, ∗∗p < 0.01, ∗∗∗p < 0.001, ∗∗∗∗p < 0.0001 compared to the control group.

## 3 Results

### 3.1 Porous properties of MCWD

**Fig. 2:**
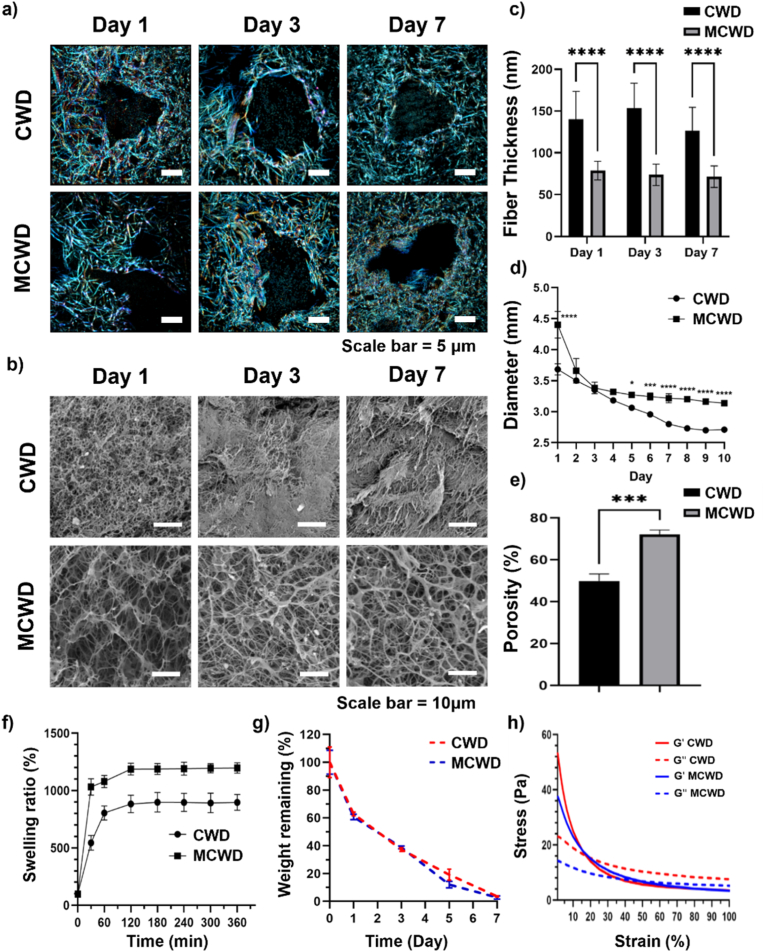
Structural characterization of CWD and MCWD as scaffolds for hADSCs. (a) STORM images showing the fluorescent staining of collagen fibers in CWD and MCWD at various time points; (b) SEM images illustrating the porous structure of CWD and MCWD; (c) Quantitative analysis of collagen fiber thickness based on STORM images (n = 10); (d) Changes in diameter of CWD and MCWD over 10 days, quantified from digital photographs; (e) Quantification of porosity from μCT analysis of CWD and MCWD (n = 3); (f) Evaluation of the swellability of CWD and MCWD to determine their exudate absorption capacities; (g) Analysis of the degradability of CWD and MCWD to assess their potential for *in vivo* absorption via biodegradation; (h) Strain-sweep analysis performed on CWD and MCWD on day 3 comparing the viscoelastic properties of CWD and MCWD.

To examine the porous properties of MCWD, the collagen fiber thickness and inner structures of MCWD were analyzed from STORM images (Fig. 2a) and SEM images (Fig. 2b), compared to those of CWD. The analysis results showed that CWD had collagen fiber thicknesses of 140.3 ± 33.3 nm on day 1, 153.6 ± 29.8 nm on day 3, and 126.7 ± 27.7 nm on day 7. In contrast, MCWD consistently showed thinner fiber thicknesses of 78.9 ± 11.2 nm, 73.7 ± 12.7 nm, and 71.6 ± 12.9 nm at the corresponding time points (Fig. 2c). The diameter of CWD decreased during culture, ranging from 3.35 ± 0.03 mm on day 3 to a final measurement of 2.71 ± 0.03 mm by day 10 (Fig. 2d). Conversely, the diameter of MCWD showed no significant reduction, remaining at 3.32 ± 0.04 mm on day 3 and measuring 3.14 ± 0.04 mm on day 10 of culture. This result indicates that MCWD experiences less shrinkage during culture compared to CWD. μCT analysis was performed to quantify the porosity of CWD and MCWD, representing the void volume of each material. The porosities of CWD and MCWD were measured to be 49.8 ± 3.4 % and 72.1 ± 2.2 %, respectively, demonstrating that MCWD possesses significantly higher porosity compared to CWD (Fig. 2e).

The water swelling ratio directly reflects porosity and the capacity to absorb exudate, which is critical for reducing the risk of infection in wound dressing [33]. Water swelling of MCWD was determined by measuring water absorption rate and comparing it with that of CWD (Fig. 2f). MCWD exhibited a significant increase in weight 30 min after water absorption, reaching 1196.9 ± 44.6 % of its lyophilized mass, whereas CWD displayed a comparatively limited swelling capacity, increasing to 897.0 ± 69.2 %.

The degradation rate of MCWD was assessed to ensure that it degrades appropriately during the tissue regeneration process without hindering wound edge approximation or causing inflammation from residual materials post-regeneration (Fig. 2g). The weight change for MCWD and CWD from day 1 to day 7 of incubation in collagenase solution showed no significant differences, indicating that their degradation rates are nearly identical. Despite the higher porosity and swelling ratio observed in MCWD compared to CWD, their degradation behavior showed no significant differences, likely due to the substantially higher collagen content in MCWD.

Strain-sweep analysis was conducted on days 3, 7, and 14 to evaluate the storage modulus and maximum strain tolerance of CWD and MCWD (Fig. 2h, Fig. S4). This evaluation was essential to determine their suitability for wound dressing applications, given the critical influence of porosity on structural integrity. Although the initial storage modulus of CWD was slightly higher than that of MCWD, the intersection points of the storage modulus (G′) and loss modulus (G″) at higher strain levels in MCWD suggest that MCWD exhibits superior maximum strain tolerance. Considering both the initial storage modulus, which reflects stiffness at low strain, and the maximum strain tolerance, indicating the capacity of the material to withstand deformation under stress, both MCWD and CWD demonstrate a well-balanced combination of structural integrity and flexibility.

Taken together, the porosity and swelling ratio of MCWD were higher than those of CWD; however, there were no significant differences in the degradation rate and deformation resistance. This is likely because MCWD is composed of a higher concentration of collagen than CWD.

### 3.2 Cellular properties of MCWD

**Fig. 3:**
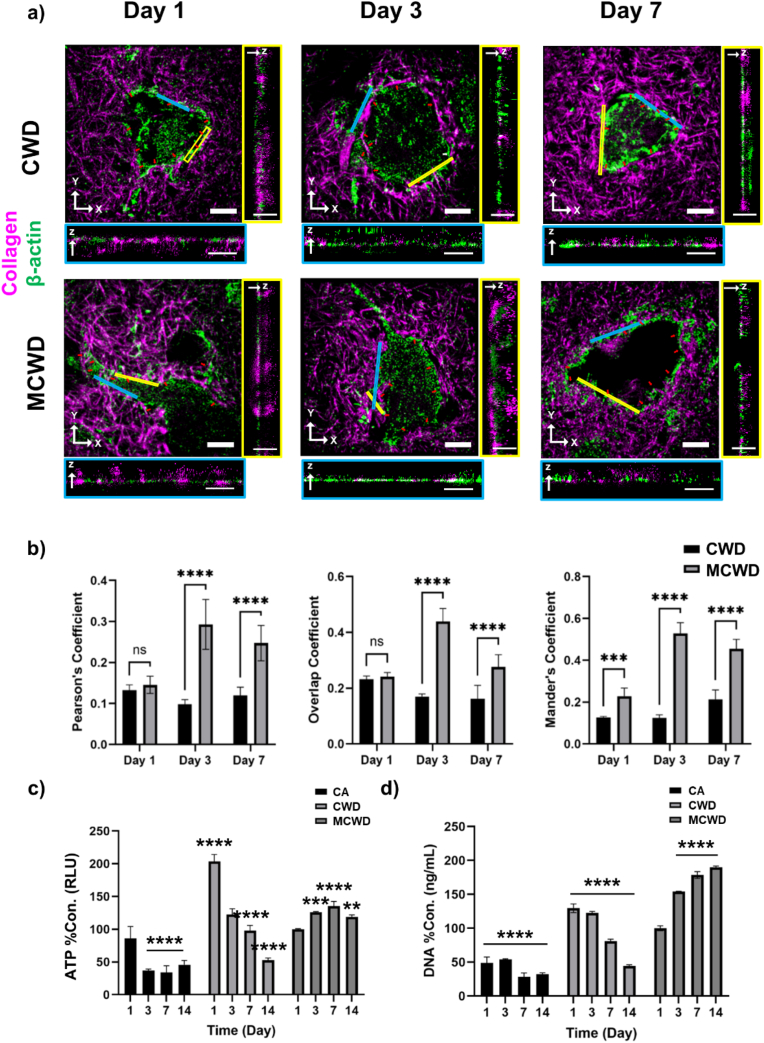
hADSC-Collagen interactions modulate cell viability and metabolic activity. (a) Two-color STORM images of hADSCs depicting β-actin (green) and collagen (purple), with cross-sectional views of the boxed regions (scale bars: 5 μm for main images, 1 μm for cross-sections); (b) Quantification of cell-collagen interactions using Pearson's, overlap, and Mander's coefficients; (c) ATP assay results indicating the metabolic activity and viability of hADSCs in CA, CWD, and MCWD, normalized to MCWD at Day 1 (n = 3); (d) DNA content analysis reflecting hADSC proliferation and viability in CA, CWD, and MCWD, normalized to MCWD at Day 1 (n = 3). (For interpretation of the references to color in this figure legend, the reader is referred to the Web version of this article.)

STORM imaging was utilized to visualize the interaction between collagen and hADSCs in response to the high porosity of MCWD (Fig. 3a). X-Y image analysis enabled the measurement of fluorescence overlap between cells and collagen, allowing for a time-dependent assessment of interaction dynamics in both CWD and MCWD. To further analyze the progression of cell-collagen interactions over time, correlation analysis of the two fluorescent channels was conducted (Fig. 3b). Multiple correlation coefficients were employed to provide a comprehensive evaluation. Pearson's coefficient, which measures the linear correlation between signal intensities, was analyzed to determine the level of intensity-based associations. The overlap coefficient, which evaluates spatial colocalization independent of intensity, assessed the degree of structural interaction. Manders' coefficient, which quantifies the fraction of one signal overlapping with another, was also examined to further characterize colocalization patterns. Analysis of these parameters revealed distinct differences between CWD and MCWD. In CWD, cell-collagen interactions remained relatively stable over time, showing minimal changes from their initial state on Day 1. In contrast, MCWD exhibited a continuous increase in interaction intensity, as indicated by the progressive rise in Pearson's, overlap, and Manders' coefficients. These findings suggest that the porous structure of MCWD facilitates sustained and dynamic interactions between hADSCs and collagen, enhancing matrix engagement over time.

To evaluate whether the enhanced porosity and cell-collagen interactions of MCWD influenced cell viability, ATP, DNA quantification, and LIVE/DEAD assays were performed (Fig. 3c, d, Fig. S5). In the ATP assay, CA exhibited low ATP activity from day 1 of culture, reflecting poorer metabolic activity compared to CWD and MCWD (Fig. 3c). CWD showed a gradual decline in ATP activity starting from day 3. In contrast, ATP activity in MCWD remained consistent over the 14-day culture period. DNA quantification results, which reflect the number of cells, showed minimal changes in MCWD during culture, whereas both CA and CWD exhibited a decline in DNA levels over time (Fig. 3d). In the LIVE/DEAD assay, dead cells (indicated by red color) were rarely observed in MCWD throughout the 14-day culture period (Fig. S5). In contrast, dead cells began to appear as early as days 1 in CA and day 7 in CWD. Notably, dead cells were primarily located within the core region of CA, indicating the formation of necrotic cores, while in CWD, dead cells were distributed throughout the entire area. These results suggest that cell survival and cellular activity are significantly enhanced in MCWD compared to CA and CWD.

### 3.3 *In vitro* evaluation of the therapeutic activity

**Fig. 4:**
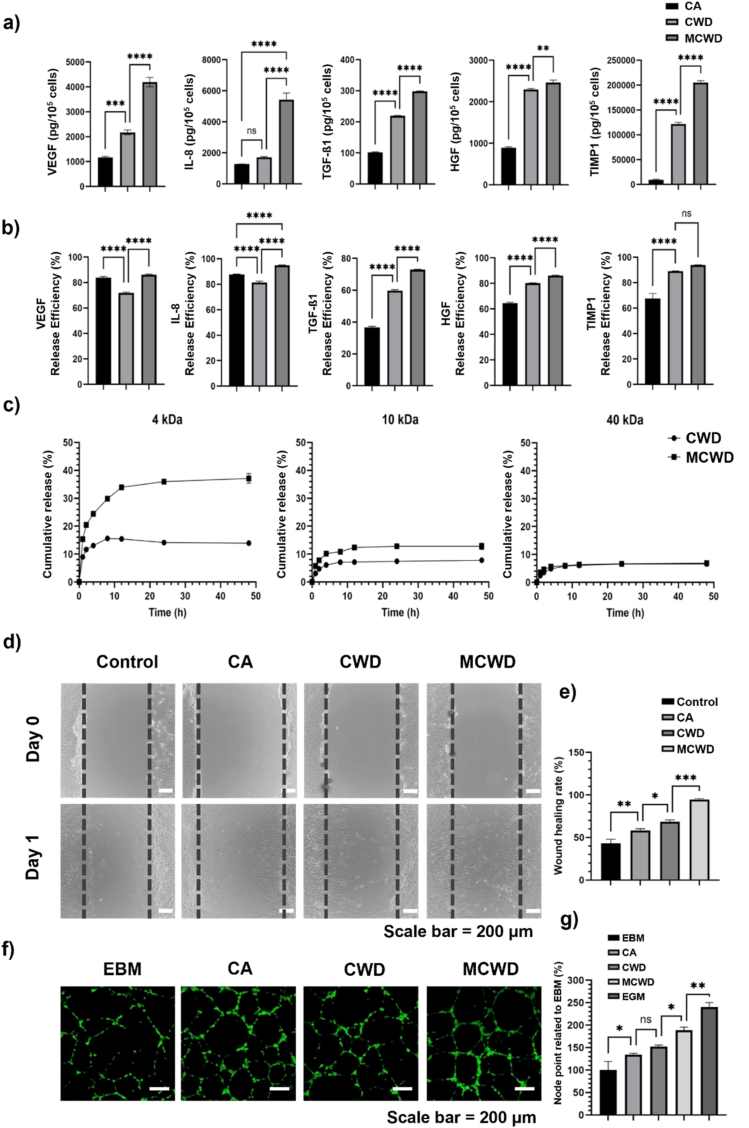
Evaluation of paracrine factor secretion profiles and their influence on the activity of wound healing-related cells in CA, CWD, and MCWD. (a) Quantification of total paracrine factor secretion across CA, CWD, and MCWD (n = 3); (b) Analysis of paracrine factor release efficiency from CA, CWD, and MCWD (n = 3); (c) Cumulative release kinetics of FITC-dextran with varying molecular weights from CA, CWD, and MCWD over 48 h (n = 3); (d) *In vitro* wound healing assay demonstrating enhanced fibroblast migration in response to paracrine signals from CA, CWD, and MCWD; (e) Quantification of wound healing rates across treatment groups (n = 3); (f) HUVEC tube formation assay assessing the angiogenic capacity of paracrine factors released from CA, CWD, and MCWD (n = 3); (g) Quantification of node points in HUVEC tube formation, indicating the degree of angiogenesis (n = 3).

The secretion profile of key paracrine factors critical for wound healing, including VEGF, IL-8, TGF-β1, HGF, and TIMP-1, were investigated across the CA, CWD, and MCWD groups (Fig. S6). To determine whether differences in porosity between MCWD and CWD influenced the release profiles of the paracrine factors, the total production of these paracrine factors—including both the quantities released into the culture medium and those retained within the respective samples—was quantified (Fig. 4a). MCWD exhibited the highest total paracrine factor production, followed by CWD and CA (Fig. 4b). Interestingly, despite the significantly lower total production of paracrine factors in CA, its release efficiency was comparable to that of CWD. This discrepancy likely stems from the absence of a scaffold in CA, which allows for more direct diffusion of secreted factors into the surrounding medium. However, in CWD, a substantial portion of the produced paracrine factors remained trapped within the scaffold, leading to a reduced release efficiency. These findings highlight the influence of scaffold architecture on paracrine factor secretion and suggest that while CWD enhances total factor production compared to CA, its release efficiency remains limited due to restricted mass transfer within the hydrogel network [34].

To further explore the differences in paracrine factor secretion between CWD and MCWD, a cumulative release analysis was performed using FITC-dextran across a range of molecular weights to identify the factors contributing to the observed variations in secretion profiles (Fig. 4c). Substances with lower molecular weights exhibited significantly faster and more pronounced release in MCWD compared to CWD, while no significant differences were observed for substances exceeding 40 kDa. This analysis indicates that MCWD facilitates a more rapid and extensive release of small molecules, such as nutrients and waste products, suggesting enhanced mass transfer capacity in MCWD compared to CWD. This enhanced capacity is likely linked to the improved cell-collagen interactions and increased cell viability observed in previous results. The molecular weights of the paracrine factors analyzed ranged from IL-8 (approximately 10 kDa), TGF-β1 (25 kDa), VEGF (30–50 kDa), TIMP-1 (30 kDa), to HGF (80 kDa). These findings suggest that the molecular weight-dependent release pattern observed in the cumulative release analysis (ranging from 4 kDa to 40 kDa) also applies to paracrine factor secretion. However, the increased paracrine factor secretion in MCWD is primarily driven by an overall increase in production levels, rather than solely by its enhanced porosity compared to CWD.

Fibroblast scratch assays and HUVEC tube formation assays were investigated to evaluate cell activation associated with granulation and angiogenesis during skin regeneration, particularly in response to the enhanced paracrine factor release observed in MCWD compared to CA and CWD. The fibroblast scratch assay results demonstrated that all treatment groups significantly enhanced fibroblast proliferation and migration rates relative to the control group, with the MCWD-treated group exhibiting the most pronounced improvement (Fig. 4d and e). Subsequently, HUVEC cell tube formation assays were conducted to assess the promotion of angiogenesis during the skin regeneration process (Fig. 4f and g). The formation of nodes, which are indicative of early angiogenic events, was significantly more pronounced in all treated groups compared to the control. Among these, MCWD exhibited the highest degree of node formation, yielding results most comparable to those observed under EGM media conditions (Fig. S7).

Prior to conducting *in vivo* experiments, a CCK-8 assays were performed to evaluate the cytotoxicity of CA, CWD, and MCWD by co-incubating them with fibroblasts for 24 h (Fig. S8a). The results showed no significant differences in cell viability compared to the control group, confirming the safety of these treatments for subsequent *in vivo* analysis.

### 3.4 *In vivo* evaluation of stem cell therapies on full-thickness wound splint model

**Fig. 5:**
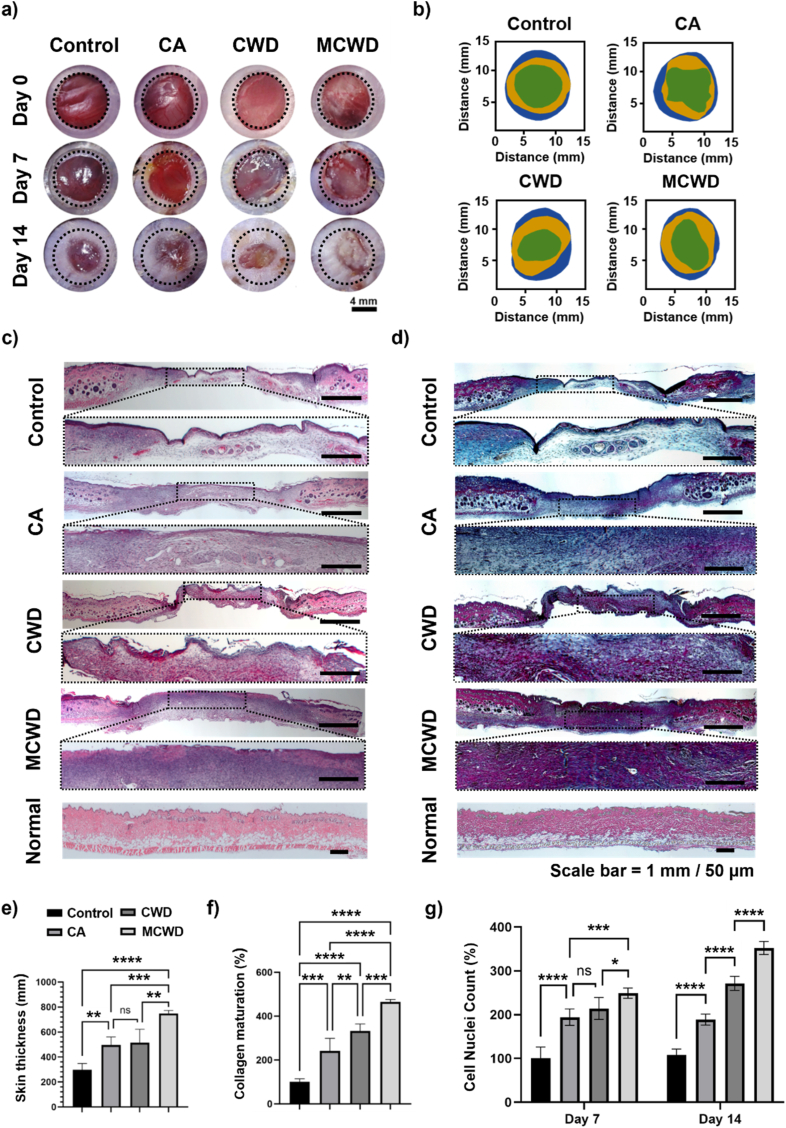
Histological assessment of an *in vivo* full-thickness wound model treated with CA, CWD, and MCWD. (a) Representative gross images showing wound healing progression; (b) Wound area tracking over 14 days, illustrating granulation-mediated closure; (c) H&E staining showing cellular infiltration, cell recruitment, and granulation tissue formation within the wound; (d) Herovici staining distinguishing immature collagen (blue) from mature collagen (red/purple), indicating the remodeling phase; (e) Quantification of skin thickness (n = 5); (f) Quantification of mature collagen deposition in wounds treated with CA, CWD, and MCWD (n = 5); (g) Quantitative analysis of cell nuclei density in the wound area, presented as the relative increase normalized to the control, reflecting the degree of cellular recruitment (n = 5). (For interpretation of the references to color in this figure legend, the reader is referred to the Web version of this article.)

An 8-mm full-thickness wound splint model was employed to evaluate the *in vivo* therapeutic efficiency of the wound dressings (Fig. 5). CA, CWD, and MCWD were applied to the silicon ring splint wound model, and wound healing progression was monitored over a 14-day period, during which no abnormal subcutaneous reactions were observed (Fig. 5a). Representative images of wound closure up to day 14 showed minimal skin wrinkling across all groups, highlighting the effectiveness of the silicon ring splint in preventing wound contraction caused by murine skin muscle movement. This model successfully created a chronic wound environment that allowed healing exclusively through tissue regeneration (Fig. 5b). As a result, wound closure was achieved solely through collagen synthesis and tissue regeneration, validating the therapeutic efficiency of stem cell therapies in a manner consistent with human wound healing dynamics (Fig. S8b).

Histological analyses, including H&E and Herovici histochemical staining, were performed to evaluate the processes of granulation and collagen maturation (Fig. 5c and d). By day 14, MCWD-treated wounds achieved a skin thickness comparable to that of the normal skin adjacent to the wound and exhibited the most substantial increase in skin thickness and granulation tissue formation across all time points (Fig. 5e). In contrast, CA- and CWD-treated wounds demonstrated less pronounced increases in skin thickness and granulation tissue formation. Herovici staining confirmed that MCWD facilitated the highest degree of collagen maturation, promoting the formation of a thicker dermal layer (Fig. 5f). Quantitative analysis of collagen maturation revealed that MCWD exhibited significantly higher levels of collagen maturation compared to CWD and CA. Furthermore, cell infiltration into the treated wound areas was quantitatively assessed by counting cell nuclei in H&E-stained images on day 7 and day 14 to evaluate the paracrine effects induced by hADSCs (Fig. 5g). At both time points, MCWD treatment resulted in a substantial increase in cell infiltration, significantly surpassing levels observed in the control, CA, and CWD.

To elucidate the cellular mechanisms underlying the histological outcomes observed in MCWD-treated wounds, RT-qPCR analysis was conducted examine the mRNA levels of key cytokines involved in the wound healing process (Fig. S9). VEGF expression showed a significant increase in the MCWD group compared to the other groups on days 3 and 7. FGF2 and TGF-β1 levels were also notably higher in the MCWD group compared to CWD, CA, and the control, with FGF2 demonstrated the most significant difference on day 7 and TGF-β1 peaking on day 14 in MCWD-treated wounds. Analysis of collagen type I and type III mRNA production further corroborated these findings. On day 7, the MCWD group exhibited elevated levels of collagen type III, while day 14, the highest levels of TGF-β1 in MCWD correlated with a significant increase in collagen type I expression. These results indicate that MCWD facilitates a dynamic modulation of cytokine and collagen expression, supporting both early and late stages of wound healing.

**Fig. 6:**
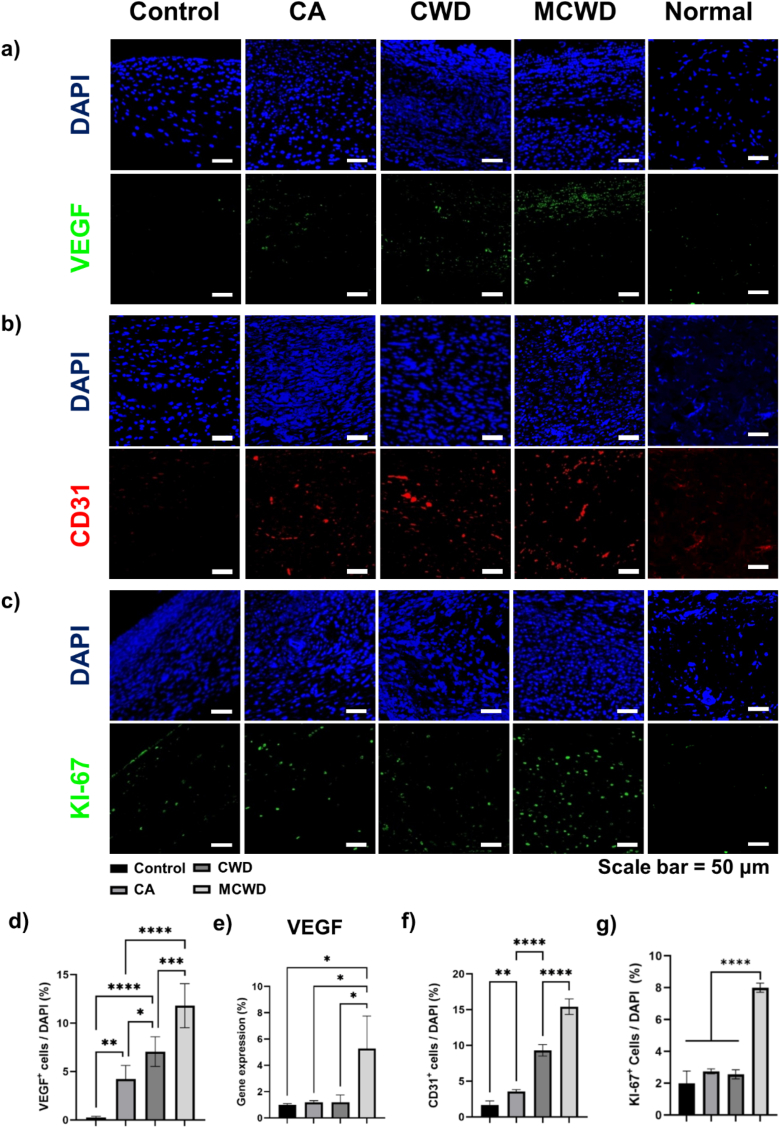
Immunofluorescence analysis of angiogenesis and cell proliferation in wound healing on day 14. (a) Representative immunofluorescence images of VEGF expression in CA, CWD, MCWD, and normal skin, showing localization and activity of the angiogenic factor; (b) Immunofluorescence staining for CD31, highlighting endothelial cell activity and neovascularization; (c) Immunofluorescence images of KI-67 expression, serving as a marker for cellular proliferation and tissue regeneration; (d) Quantitative analysis of VEGF^+^ cells, providing a detailed assessment of angiogenic activity; (e) *In vivo* qPCR quantification of VEGF mRNA levels during wound healing (n = 3); (f) Quantitative analysis of CD31^+^ cells, reflecting the extent of endothelial cell recruitment and capillary formation; (g) Quantification of KI-67^+^ cells, assessing proliferative capacity.

To evaluate the extent of vascular regeneration via angiogenesis, immunofluorescence imaging was performed to observe key markers of vascular regeneration—VEGF and CD31—along with cellular proliferation assessed via Ki-67 staining on day 14 (Fig. 6a–c). The analysis showed that MCWD-treated wounds exhibited the highest percentage of VEGF^+^ cells, significantly surpassing CWD, CA, and the control groups (Fig. 6d). This finding was corroborated by *in vivo* qPCR analysis, which confirmed that VEGF expression levels were highest in the MCWD group (Fig. 6e). Angiogenesis, driven by VEGF, was further assessed using CD31 staining, a robust marker of early angiogenesis and vascular remodeling. The results demonstrated a pronounced elevation in CD31^+^ cell expression in the MCWD group compared to the CWD, CA, and the control groups (Fig. 6f). Additionally, Ki-67 immunofluorescence staining, an established indicator of cellular proliferation, showed a markedly increased expression of Ki-67^+^ cells in MCWD-treated wounds, significantly exceeding levels observed in the other groups (Fig. 6g).

## 4 Discussion

A key distinction between MCWD and CWD is the regulation of collagen fibrillogenesis, which significantly impacts collagen fiber thickness and overall mechanical properties. STORM, SEM, and shrinkage analyses revealed that MCWD effectively inhibits long-term fibrillogenesis, thereby preventing the self-assembly of collagen fibers into thick microbundles that would otherwise form a compact structure with reduced porosity (Fig. 2). The increased porosity and enhanced surface-to-volume ratio of MCWD not only improve its efficacy as a wound dressing but also confer advantages in swelling capacity and degradation rate compared to CWD. In terms of mechanical performance, MCWD demonstrates a significant increase in maximum strain tolerance, showing nearly a threefold improvement compared to CWD. This enhancement highlights the superior flexibility of MCWD and its resistance to deformation under external forces—properties essential for wound environments characterized by constant mechanical stress and the need to accommodate skin elasticity. These findings align with previous studies emphasizing deformation resistance as a critical factor in ensuring the long-term therapeutic efficacy of wound dressings [27]. While MCWD is not self-adhesive, its strong hydrophilicity creates an optimal environment for cell activity and tissue regeneration while minimizing adhesion to the wound site. This characteristic reduces trauma during dressing changes and enhances patient comfort [35]. Additionally, incorporating an adhesive border would ensure that the dressing remains securely in place while allowing for easy removal with minimal damage to the wound area, thereby improving overall usability [36].

The strong interactions between hADSCs and collagen observed in STORM imaging indicate that the micro-fragmented collagen hydrogel creates an optimal environment for hADSCs, crucial for maintaining cell viability and enhancing cellular activation (Fig. 3). The increased porosity of MCWD likely facilitates more efficient exchange of essential nutrients and waste products, thereby improving hADSCs viability and strengthening cell-collagen interactions. This favorable microenvironment is critical for enhancing cellular activation and paracrine factor synthesis, as supported by previous studies [37]. Cytotoxicity analysis underscored the significant influence of porosity on cell viability and metabolic activity. While CWD initially showed higher ATP and DNA concentrations−attributed to the rapid stabilization of the collagen hydrogel through fibrillogenesis−continuous fibrillogenesis in CWD hindered mass transfer, leading to nutrient deprivation, hypoxia, and apoptosis, particularly in the core regions of the scaffold. In contrast, the enhanced porosity of MCWD effectively supported nutrient and waste exchange, maintaining higher cell viability and metabolic activity over time.

The synergistic interaction of VEGF, TGF-β1, HGF, and IL-8 is crucial for orchestrating an effective wound healing response [[38], [39], [40]]. VEGF and IL-8 facilitate angiogenesis and enhance vascular permeability, ensuring adequate nutrient and immune cell infiltration at the wound site. TGF-β1 induces extracellular matrix synthesis, modulates the immune response, and promotes fibroblast activity, contributing to the structural integrity and remodeling of healing tissue. Meanwhile, HGF drives cellular proliferation, migration, and tissue regeneration. This coordinated action ensures a balanced and efficient wound healing process, resulting in well-vascularized, structurally robust, and functionally restored tissue. The paracrine effects of hADSCs were significantly enhanced in MCWD, with the secreted factors playing a pivotal role in promoting cellular activity (Fig. 4d–g). Notably, hADSCs within MCWD stimulated the release of essential growth factors, including VEGF, HGF, and FGF2. As reported in previous studies, the paracrine effects of hADSCs induced the release of critical growth factors, such as VEGF, HGF, and FGF2, which facilitated the proliferation, migration, and activation of fibroblasts and HUVECs. *In vivo* results demonstrated a 1.5-fold increase in skin thickness and a nearly fourfold expansion in vascular structures compared to controls, leading to enhanced collagen deposition, follicular rejuvenation, and neovascularization [41]. The upregulation of fibroblast activation underscores the superior efficacy of MCWD in promoting wound repair. This effect is primarily driven by the enhanced paracrine signaling of hADSCs encapsulated within MCWD, facilitating the sustained release of key growth factors into the surrounding environment. These secreted factors, including VEGF, FGF2, HGF, PDGF, TGF-β1, and IL-8, play pivotal roles in fibroblast proliferation, migration, and extracellular matrix remodeling—essential processes in wound healing [3,42]. The robust paracrine signaling in MCWD was further demonstrated in the HUVEC tube formation assay, where increased node formation indicated a strong angiogenic response. Early angiogenesis is critical for supplying nutrients and oxygen to regenerating tissue, thereby accelerating the overall wound healing process [43]. These findings emphasize the importance of paracrine signaling mechanisms in stem cell-based wound healing therapies and the potential of MCWD as an effective biomaterial for enhancing tissue regeneration.

Histological analysis provided further evidence of the therapeutic potential of MCWD in promoting wound healing (Fig. 5). The silicon ring splint successfully suppressed wound contraction caused by dorsal muscle movement for up to 14 days (Fig. S8b). This stabilization minimized inter-group variability in wound closure rates, enabling a more precise histological assessment of the healing efficiency of regenerated skin tissue. H&E and Herovici staining revealed enhanced skin regeneration and advanced collagen maturation facilitated by MCWD, suggesting that it supports long-term skin tissue stability and functionality. The transition from collagen type III in early granulation tissue to mature collagen type I is a critical process for restoring structural integrity and functional resilience during the remodeling phase [44]. Notably, MCWD accelerated this collagen maturation process, significantly reducing the time required for skin restoration while ensuring a stable progression through the remodeling phase. This stability is essential for minimizing the risk of adverse fibrotic responses [45]. The enhanced cellular recruitment observed in MCWD-treated wounds further highlights the strong paracrine influence of hADSCs encapsulated within the hydrogel. This *in vivo* observation is consistent with previous *in vitro* findings, which demonstrated the superior secretion efficacy of MCWD in promoting cell activation. Specifically, the sustained release of key growth factors such as VEGF, bFGF, and PDGF-AA, along with chemokines secreted by hADSCs, has been shown to drive enhanced cellular migration, a crucial step in wound healing [46]. These findings underscore the translational potential of MCWD in accelerating tissue regeneration through both structural support and biochemical signaling.

*In vivo* RT-qPCR analysis provided valuable insights into the differential expression of key cytokines and collagen types during the wound healing process across the control, CA, CWD, and MCWD groups (Fig. S9). Notably, VEGF and FGF2 expression levels were significantly elevated in the MCWD group on day 3 and day 7 compared to the other groups. These growth factors act as critical regulators of angiogenesis and have been shown to synergistically enhance endothelial cell function and vascularization [38]. Specifically, VEGF primarily promotes endothelial cell activity and vascular permeability, while FGF2 supports endothelial cell proliferation, differentiation, and vessel stabilization. Their cooperative interaction is known to potentiate VEGF expression and receptor sensitivity, thereby driving robust angiogenesis and improving wound healing outcomes [47]. The early upregulation of VEGF and FGF2 in the MCWD group facilitated rapid vascularization. FGF2 and TGF-β1, cytokines that promote fibroblast proliferation and differentiation, also exhibited significantly higher levels in the MCWD group compared to the CWD, CA, and control groups. The substantial difference in FGF2 levels observed on day 7 underscores its pivotal role in early fibroblast proliferation and wound healing. Additionally, TGF-β1 levels peaked on day 14 in the MCWD group, highlighting its role in the later stages of healing by facilitating fibroblasts differentiation into myofibroblasts—a process essential for matrix remodeling and wound contraction [48]. Further analysis of collagen type I and collagen type III mRNA production corroborated these findings. On day 7, the MCWD group exhibited elevated levels of collagen type III, indicative of active fibroblast proliferation driven by FGF2. By day 14, the highest levels of TGF-β1 in the MCWD group correlated with increased collagen type I expression, suggesting that TGF-β1 upregulation promoted myofibroblast differentiation and facilitated efficient matrix remodeling.

Immunofluorescence analysis provided compelling evidence of the superior efficacy of MCWD in promoting critical aspects of wound healing, particularly angiogenesis and cell proliferation (Fig. 6). The MCWD group demonstrated the highest percentage of VEGF^+^ cells, indicating that the micro-fragmented collagen hydrogel scaffold effectively sustained VEGF activity—a crucial factor known to promote angiogenesis [47]. This sustained VEGF presence facilitated the recruitment and proliferation of endothelial cells, thereby initiating and maintaining the angiogenic process. Additionally, the marked increase in CD31^+^ cell expression in the MCWD-treated wounds indicates robust early angiogenesis and vascular remodeling. CD31, a well-established marker for endothelial cells, plays a critical role in the formation of new blood vessel formation. Furthermore, analysis of KI-67, a widely recognized marker of cellular proliferation, validated the enhanced therapeutic efficacy of MCWD group, underscoring the direct relationship between improved vascularization and increased cellular proliferation.

This study highlights that MCWD represents a significant advancement over conventional collagen hydrogel-based wound dressings by effectively overcoming the limitations of low porosity, which critically hampers the therapeutic potential of stem cell therapy in wound healing. The increased porosity of MCWD markedly enhanced the survival and activation of encapsulated hADSCs, leading to elevated production of paracrine factors and extended therapeutic efficacy. However, further research is required to elucidate the mechanisms underlying this enhanced therapeutic effect. Previous studies have demonstrated that various microenvironmental scaffold properties—such as porosity, stiffness, alignment, and biochemical affinity—collectively influence key intracellular signaling pathways in hADSCs, including the Rho/ROCK pathway, which regulates essential cellular processes such as proliferation, differentiation, and activation [49,50]. It is plausible that the enhanced interactions between cells and collagen within MCWD contributed to the observed increase in paracrine factor synthesis associated with wound healing. Future investigations should focus on examining how these modulated cell-collagen interaction dynamics in MCWD influence the production and secretion of paracrine factors. Furthermore, a direct comparison between MCWD and commercially available scaffolds would enable a more comprehensive evaluation of its therapeutic potential. Assessing MCWD alongside clinically established biomaterials with distinct structural and biochemical properties could clarify its effectiveness in stem cell-mediated wound healing. Such analyses would not only enhance its clinical relevance but also inform the rational design of next-generation wound dressings to improve therapeutic outcomes. Additionally, assessing the therapeutic effect of micro-fragmented collagen hydrogel alone was challenging, as it remained in a solution state and failed to form a stable hydrogel in the absence of hADSCs. Similarly, our previous research on a critical limb ischemia model demonstrated that micro-fragmented collagen hydrogel alone did not provide significant therapeutic benefits, as it rapidly dispersed upon injection [15]. Specifically, injection of the hydrogel without hADSCs failed to improve blood perfusion or limb salvage compared to the PBS-injected group, highlighting the necessity of cellular components for its efficacy. These findings reinforce the notion that hADSCs play a crucial role in enhancing the therapeutic potential of the hydrogel by supporting tissue repair and providing a stable microenvironment for cell retention and activity. However, its effectiveness in wound healing remains uncertain, as tissue-specific factors may yield different outcomes. Such insights would be invaluable for optimizing the design of next-generation wound dressings, enabling improved therapeutic outcomes through precise control over stem cell behavior.

## 5 Conclusion

This study introduces an advanced micro-fragmented collagen hydrogel wound dressing (MCWD) that demonstrates superior therapeutic efficacy compared to traditional stem cell therapies, including cell aggregates (CA) and hydrogel wound dressings (CWD). The high porosity of MCWD facilitates enhanced material exchange, supporting the proliferation, survival, and paracrine factor synthesis of hADSCs while improving their delivery to the wound site. As a result, significant improvements in cell recruitment, angiogenesis, collagen synthesis, and maturation were observed, collectively enhancing overall wound healing efficacy. These findings highlight the potential of MCWD as an innovative and highly effective material for chronic wound treatment. By optimizing stem cell therapy outcomes, MCWD represents a significant advancement in regenerative medicine, offering promising implications for the clinical management of chronic wounds.

## CRediT authorship contribution statement

**Changgi Hong:** Writing – original draft, Visualization, Validation, Software, Resources, Methodology, Formal analysis, Data curation, Conceptualization. **Youngseop Lee:** Visualization, Software, Methodology, Data curation. **Haeun Chung:** Resources, Methodology. **Dongwoo Kim:** Methodology, Data curation. **Jeongmin Kim:** Visualization, Software, Resources, Methodology, Data curation. **Jong-Wan Kim:** Resources. **Kangwon Lee:** Validation, Supervision, Project administration, Investigation, Funding acquisition. **Sang-Heon Kim:** Writing – review & editing, Visualization, Validation, Supervision, Resources, Project administration, Methodology, Investigation, Funding acquisition, Formal analysis, Data curation, Conceptualization.

## Declaration of competing interest

The authors declare that they have no known competing financial interests or personal relationships that could have appeared to influence the work reported in this paper.

## Data Availability

Data will be made available on request.

## References

[1] Dehkordi A.N., Babaheydari F.M., Chehelgerdi M., Dehkordi S.R. (2019). Skin tissue engineering: wound healing based on stem-cell-based therapeutic strategies. Stem Cell Res. Ther..

[2] Kucharzewski M., Rojczyk E., Wilemska-Kucharzewska K., Wilk R., Hudecki J., Los M.J. (2019). Novel trends in application of stem cells in skin wound healing. Eur. J. Pharmacol..

[3] Wang M., Wang C.G., Chen M., Xi Y.W., Cheng W., Mao C., Xu T.Z., Zhang X.X., Lin C., Gao W.Y., Guo Y., Lei B. (2019). Efficient angiogenesis-based diabetic wound healing/skin reconstruction through bioactive antibacterial adhesive ultraviolet shielding nanodressing with exosome release. ACS Nano.

[4] Ul Hassan W., Greiser U., Wang W.X. (2014). Role of adipose-derived stem cells in wound healing. Wound Repair Regen..

[5] McKee C., Chaudhry G.R. (2017). Advances and challenges in stem cell culture. Colloid Surface B.

[6] Kim W., Gwon Y., Park S., Kim H., Kim J. (2023). Therapeutic strategies of three-dimensional stem cell spheroids and organoids for tissue repair and regeneration. Bioact. Mater..

[7] Nilforoushzadeh M.A., Yazdi M.K., Ghavami S.B., Farokhimanesh S., Amirabad L.M., Zarrintaj P., Saeb M.R., Hamblin M.R., Zare M., Mozafari M. (2020). Mesenchymal stem cell spheroids embedded in an injectable thermosensitive hydrogel: an in situ drug formation platform for accelerated wound healing. Acs Biomater Sci Eng.

[8] Li Y.Z., Zhang J., Wang C.Z., Jiang Z.W., Lai K.C., Wang Y., Yang G.L. (2023). Porous composite hydrogels with improved MSC survival for robust epithelial sealing around implants and M2 macrophage polarization. Acta Biomater..

[9] Shojaei F., Rahmati S., Dehkordi M.B. (2019). A review on different methods to increase the efficiency of mesenchymal stem cell-based wound therapy. Wound Repair Regen..

[10] Baldari S., Di Rocco G., Piccoli M., Pozzobon M., Muraca M., Toietta G. (2017). Challenges and strategies for improving the regenerative effects of mesenchymal stromal cell-based therapies. Int. J. Mol. Sci..

[11] Kim S.W., Seo I., Hyun J., Eom J., Um S.H., Bhang S.H. (2023). Fibronectin-Enriched interface using a spheroid-converged cell sheet for effective wound healing. Acs Appl Mater Inter.

[12] Xu Q., Sigen A., Gao Y.S., Guo L.R., Creagh-Flynn J., Zhou D.Z., Greiser U., Dong Y.X., Wang F.G., Tai H.Y., Liu W.G., Wang W., Wang W.X. (2018). A hybrid injectable hydrogel from hyperbranched PEG macromer as a stem cell delivery and retention platform for diabetic wound healing. Acta Biomater..

[13] Liu Z.M., Tang M.L., Zhao J.P., Chai R.J., Kang J.H. (2018). Looking into the future: toward advanced 3D biomaterials for stem-cell-based regenerative medicine. Adv Mater.

[14] Feng Q., Li D.G., Li Q.T., Cao X.D., Dong H. (2022). Microgel assembly: fabrication, characteristics and application in tissue engineering and regenerative medicine. Bioact. Mater..

[15] Chung H., Choi J.K., Hong C., Lee Y., Hong K.H., Oh S.J., Kim J., Song S.C., Kim J.W., Kim S.H. (2024). A micro-fragmented collagen gel as a cell-assembling platform for critical limb ischemia repair. Bioact. Mater..

[16] Feng Q., Gao H.C., Wen H.J., Huang H.H., Li Q.T., Liang M.H., Liu Y., Dong H., Cao X.D. (2020). Engineering the cellular mechanical microenvironment to regulate stem cell chondrogenesis: insights from a microgel model. Acta Biomater..

[17] Daly A.C., Riley L., Segura T., Burdick J.A. (2020). Hydrogel microparticles for biomedical applications. Nat. Rev. Mater..

[18] Lee J., Oh S.J., An S.H., Kim W.D., Kim S.H. (2020). Machine learning-based design strategy for 3D printable bioink: elastic modulus and yield stress determine printability. Biofabrication.

[19] Chen S.W., Zhang Q., Nakamoto T., Kawazoe N., Chen G.P. (2014). Highly active porous scaffolds of collagen and hyaluronic acid prepared by suppression of polyion complex formation. J. Mater. Chem. B.

[20] Xin X.J., Borzacchiello A., Netti P.A., Ambrosio L., Nicolais L. (2004). Hyaluronic-acid-based semi-interpenetrating materials. J Biomat Sci-Polym E.

[21] Foty R. (2011). A simple hanging drop cell culture protocol for generation of 3D spheroids. J. Vis. Exp..

[22] He J., Zhang N.H., Zhu Y., Jin R.R., Wu F. (2021). MSC spheroids-loaded collagen hydrogels simultaneously promote neuronal differentiation and suppress inflammatory reaction through PI3K-Akt signaling pathway. Biomaterials.

[23] Zhang Z.K., Li Z., Li Y., Wang Y.Y., Yao M.H., Zhang K., Chen Z.Y., Yue H., Shi J.J., Guan F.X., Ma S.S. (2021). Sodium alginate/collagen hydrogel loaded with human umbilical cord mesenchymal stem cells promotes wound healing and skin remodeling. Cell Tissue Res..

[24] Mirshekar M., Afkhami H., Razavi S., Jazi F.M., Darban-Sarokhalil D., Ohadi E., Nezhad M.M., Karimi R. (2023). Potential antibacterial activity and healing effect of topical administration of bone marrow and adipose mesenchymal stem cells encapsulated in collagen-fibrin hydrogel scaffold on full-thickness burn wound infection caused by. Burns.

[25] Lee Y.S., Lee Y.H., Lee M.C., Koo D., Kim D., Kim H., Lee K.W., Kim J. (2023). STORM imaging buffer with a refractive index matched to standard immersion oil. Acs Photonics.

[26] Bolte S., Cordelières F.P. (2006). A guided tour into subcellular colocalization analysis in light microscopy. J Microsc-Oxford.

[27] Hong C., Chung H., Lee G., Kim D., Jiang Z., Kim S.H., Lee K. (2024). Remendable cross-linked alginate/gelatin hydrogels incorporating nanofibers for wound repair and regeneration. Biomacromolecules.

[28] Hong C., Chung H., Lee G., Kim C., Kim D., Oh S.J., Kim S.H., Lee K. (2023). Hydrogel/nanofiber composite wound dressing optimized for skin layer regeneration through the mechanotransduction-based microcellular environment. ACS Appl. Bio Mater..

[29] Lotz C., Schmid F.F., Oechsle E., Monaghan M.G., Walles H., Groeber-Becker F. (2017). Cross-linked collagen hydrogel matrix resisting contraction to facilitate full-thickness skin equivalents. Acs Appl Mater Inter.

[30] Kim J., Kim Y.M., Song S.C. (2023). One-step preparation of an injectable hydrogel scaffold system capable of sequential dual-growth factor release to maximize bone regeneration. Adv Healthc Mater.

[31] Kim H., Joo Y., Kook Y.M., Tran N.L., Kim S.H., Lee K., Oh S.J. (2022). On-demand local immunomodulation via epigenetic control of macrophages using an inflammation-responsive hydrogel for accelerated wound healing. ACS Appl. Mater. Interfaces.

[32] Suarez-Arnedo A., Torres Figueroa F., Clavijo C., Arbelaez P., Cruz J.C., Munoz-Camargo C. (2020). An image J plugin for the high throughput image analysis of in vitro scratch wound healing assays. PLoS One.

[33] Liang Y.P., He J.H., Guo B.L. (2021). Functional hydrogels as wound dressing to enhance wound healing. ACS Nano.

[34] Thai V.L., Ramos-Rodriguez D.H., Mesfin M., Leach J.K. (2023). Hydrogel degradation promotes angiogenic and regenerative potential of cell spheroids for wound healing. Mater Today Bio.

[35] Obagi Z., Damiani G., Grada A., Falanga V. (2019). Principles of wound dressings: a review. Surg. Technol. Int..

[36] Op 't Veld R.C., Walboomers X.F., Jansen J.A., Wagener F. (2020). Design considerations for hydrogel wound dressings: strategic and molecular advances. Tissue Eng Part B Rev.

[37] Jooybar E., Abdekhodaie M.J., Karperien M., Mousavi A., Alvi M., Dijkstra P.J. (2020). Developing hyaluronic acid microgels for sustained delivery of platelet lysate for tissue engineering applications. Int. J. Biol. Macromol..

[38] Barrientos S., Stojadinovic O., Golinko M.S., Brem H., Tomic-Canic M. (2008). Growth factors and cytokines in wound healing. Wound Repair Regen..

[39] Behm B., Babilas P., Landthaler M., Schreml S. (2012). Cytokines, chemokines and growth factors in wound healing. J Eur Acad Dermatol.

[40] Choi J., Choi W., Joo Y., Chung H., Kim D., Oh S.J., Kim S.H. (2021). FGF2-primed 3D spheroids producing IL-8 promote therapeutic angiogenesis in murine hindlimb ischemia. NPJ Regen. Med..

[41] Wu X.Y., Zhu H.F., Che J.Y., Xu Y., Tan Q., Zhao Y.J. (2023). Stem cell niche-inspired microcarriers with ADSCs encapsulation for diabetic wound treatment. Bioact. Mater..

[42] Sterodimas A., de Faria J., Nicaretta B., Pitanguy I. (2010). Tissue engineering with adipose-derived stem cells (ADSCs): current and future applications. J Plast Reconstr Aes.

[43] Cai Y., Li J.Y., Jia C.S., He Y.F., Deng C.L. (2020). Therapeutic applications of adipose cell-free derivatives: a review. Stem Cell Res. Ther..

[44] Kwon J.W., Savitri C., An B., Yang S.W., Park K. (2023). Mesenchymal stem cell-derived secretomes-enriched alginate/extracellular matrix hydrogel patch accelerates skin wound healing. Biomater. Res..

[45] Younesi F.S., Miller A.E., Barker T.H., Rossi F.M.V., Hinz B. (2024). Fibroblast and myofibroblast activation in normal tissue repair and fibrosis. Nat. Rev. Mol. Cell Biol..

[46] Cai Y., Li J., Jia C., He Y., Deng C. (2020). Therapeutic applications of adipose cell-free derivatives: a review. Stem Cell Res. Ther..

[47] Bao P., Kodra A., Tomic-Canic M., Golinko M.S., Ehrlich H.P., Brem H. (2009). The role of vascular endothelial growth factor in wound healing. J. Surg. Res..

[48] Barrientos S., Brem H., Stojadinovic O., Tomic-Canic M. (2014). Clinical application of growth factors and cytokines in wound healing. Wound Repair Regen..

[49] Guo X., Wang X., Tang H., Ren Y., Li D., Yi B., Zhang Y. (2022). Engineering a mechanoactive fibrous substrate with enhanced efficiency in regulating stem cell tenodifferentiation. ACS Appl. Mater. Interfaces.

[50] Molley T.G., Hung T.T., Kilian K.A. (2022). Cell-laden gradient microgel suspensions for spatial control of differentiation during biofabrication. Adv Healthc Mater.

