# Supplementary material for: Micro-fragmented collagen hydrogel wound dressing: Enhanced porosity facilitates elevated stem cell survival and paracrine effects for accelerated wound maturation
Source: Mater Today Bio. 2025 Mar 22;32:101678. doi: 10.1016/j.mtbio.2025.101678 (PMC11986482; doi:10.1016/j.mtbio.2025.101678)
Supplement: Multimedia component 1 [file mmc1.docx]

**Micro-fragmented Collagen Hydrogel Wound Dressing: Enhanced Porosity Facilitates Elevated Stem Cell Survival and Paracrine Effects for Accelerated Wound Maturation**

**Changgi Hong**‡**^a,b^, Youngseop Lee^b,d^, Haeun Chung^a,c^, Dongwoo Kim^b,d^, Jeongmin Kim^b,d^, Jong-Wan Kim^e^ , Kangwon Lee*^b,d^, Sang-Heon Kim*^a,c^**

^a^ *Center for Biomaterials, Biomedical Research Institute, Korea Institute of Science and Technology (KIST), 02792, Seoul, Republic of Korea*

^b^ *Department of Applied Bioengineering, Graduate School of Convergence Science and Technology, Seoul National University, Seoul, 08826, Republic of Korea*

^c^ *Division of Bio-Medical Science and Technology, KIST School, Korea University of Science and Technology, Seoul, 02792, Republic of Korea*

^d^ *Research Institute for Convergence Science, Seoul National University, Seoul 08826, Republic of Korea*

^e^ *S.Biomedics Co., Ltd., Seoul, 04797, Republic of Korea*

*‡ indicates first authors*

** indicates corresponding authors*

*Email: Kangwon Lee (*[*kangwonlee@snu.ac.kr*](mailto:kangwonlee@snu.ac.kr)*), Sang-Heon Kim (*[*skimbrc@kist.re.kr*](mailto:skimbrc@kist.re.kr)*)*


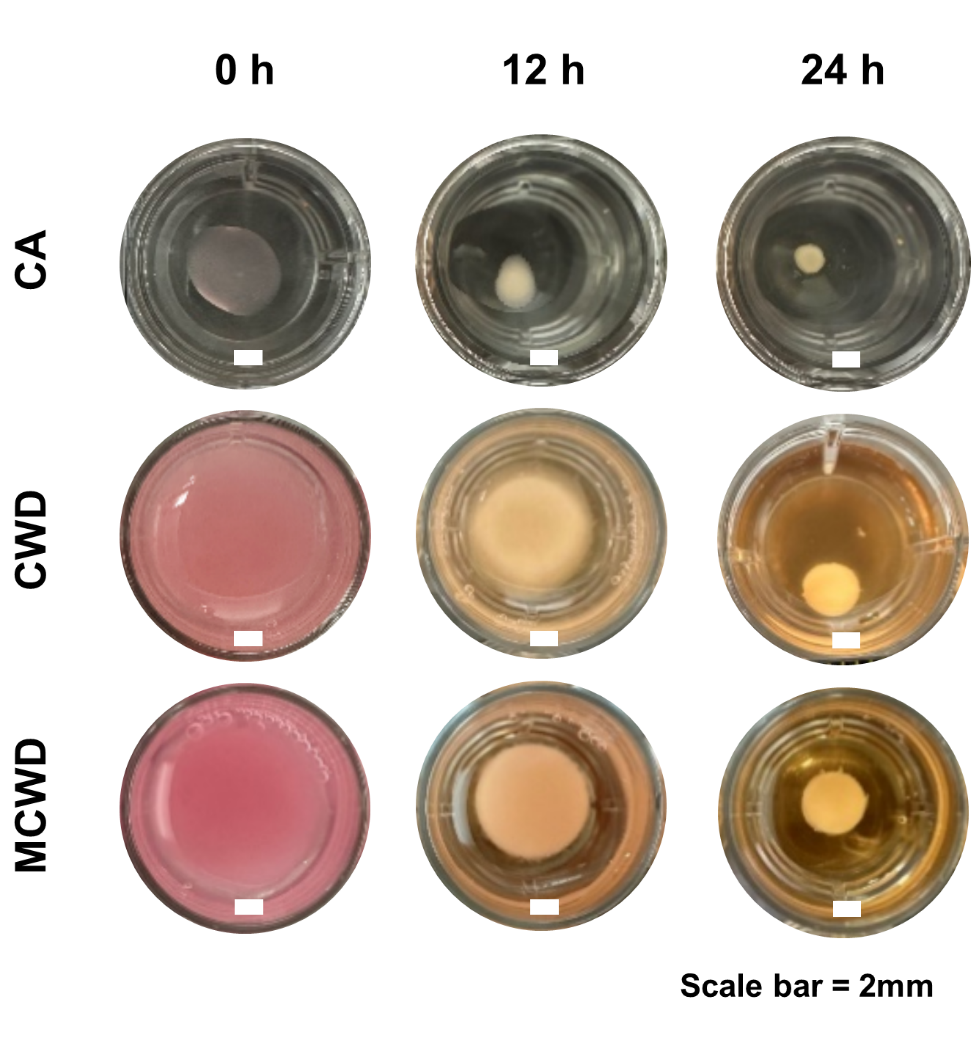


**Figure S1.** Representative photographs of cell aggregate (CA), collagen hydrogel wound dressing (CWD), and micro-fragmented collagen hydrogel wound dressing (MCWD).


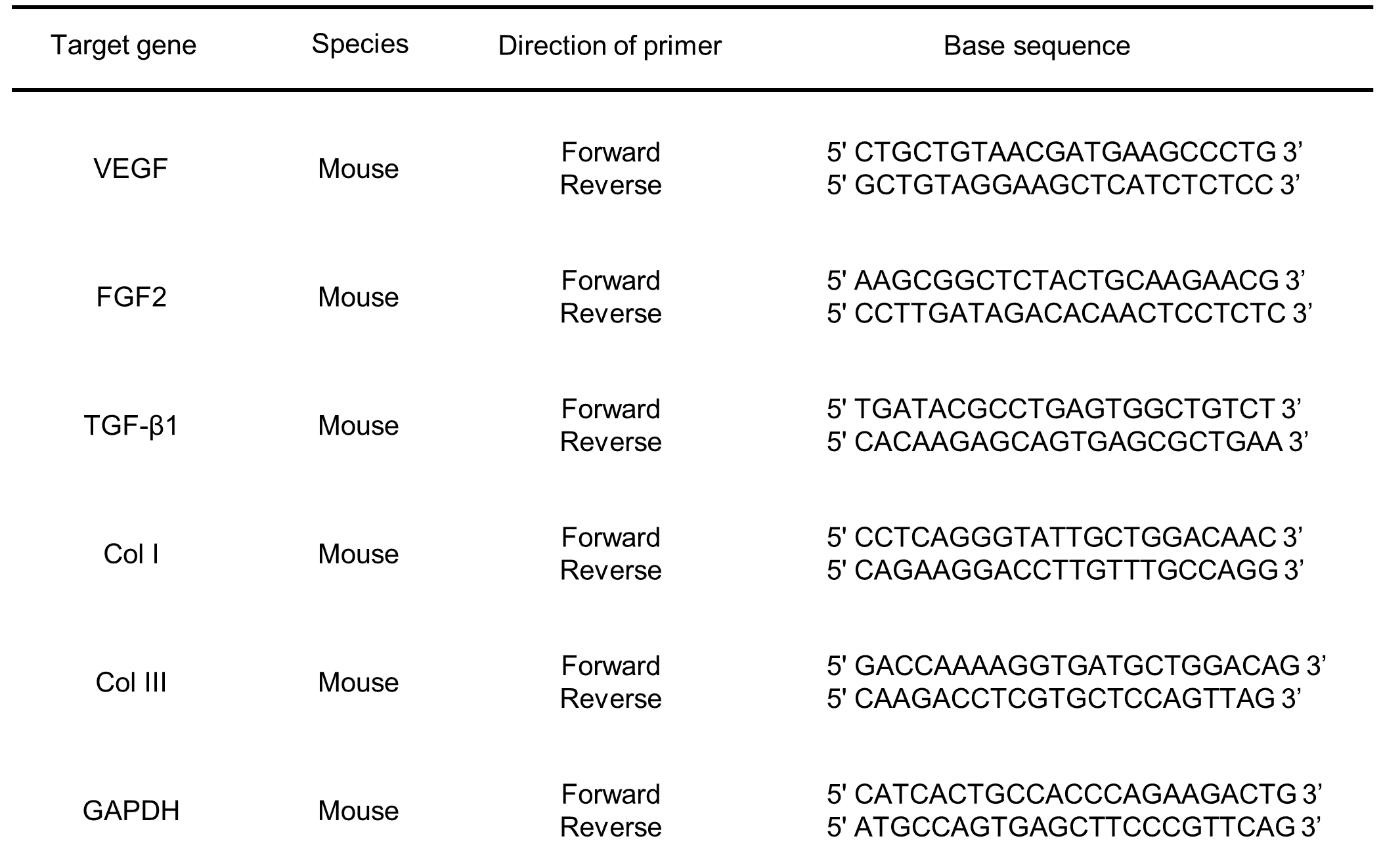


**Figure S2.** Sequences of forward and reverse primers used in RT-qPCR analysis


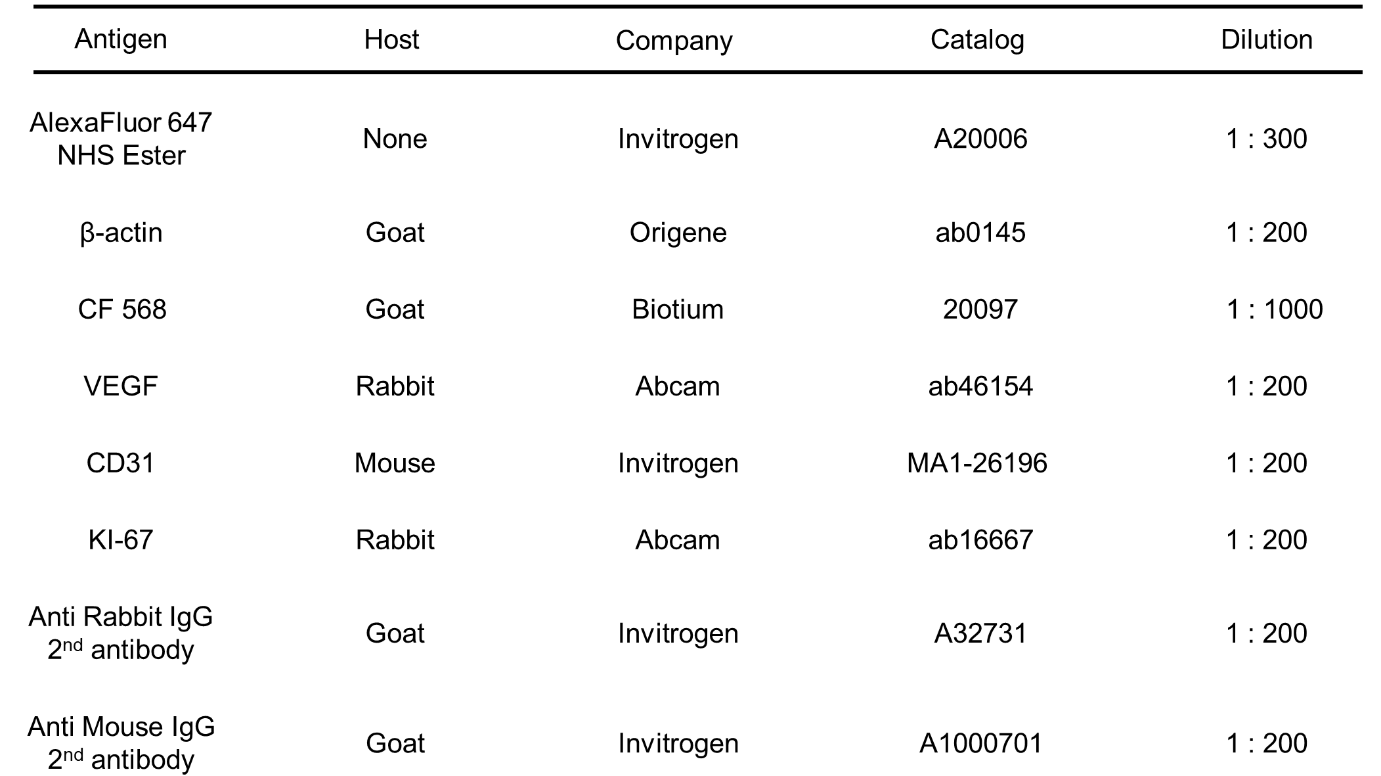


**Figure S3.** List of antibodies used in the study


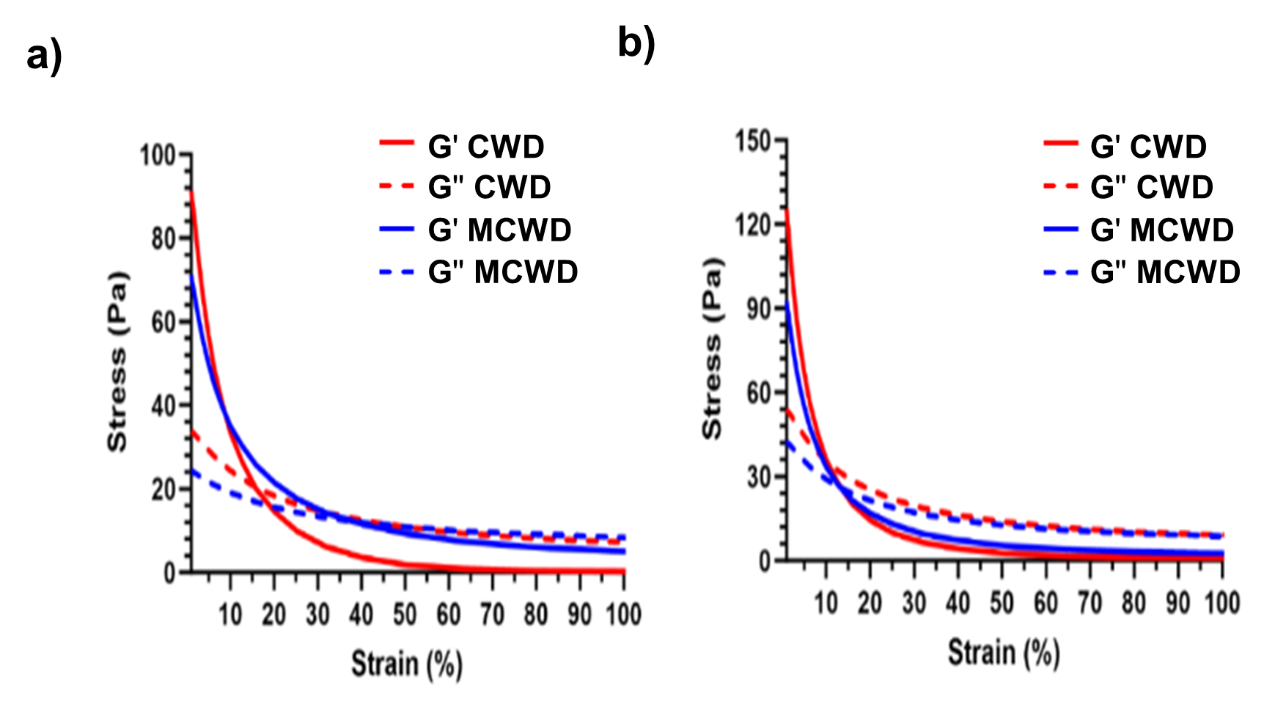


**Figure S4.** Strain-sweep analysis performed on CWD and MCWD on day 3 and day 14 comparing the viscoelastic properties.


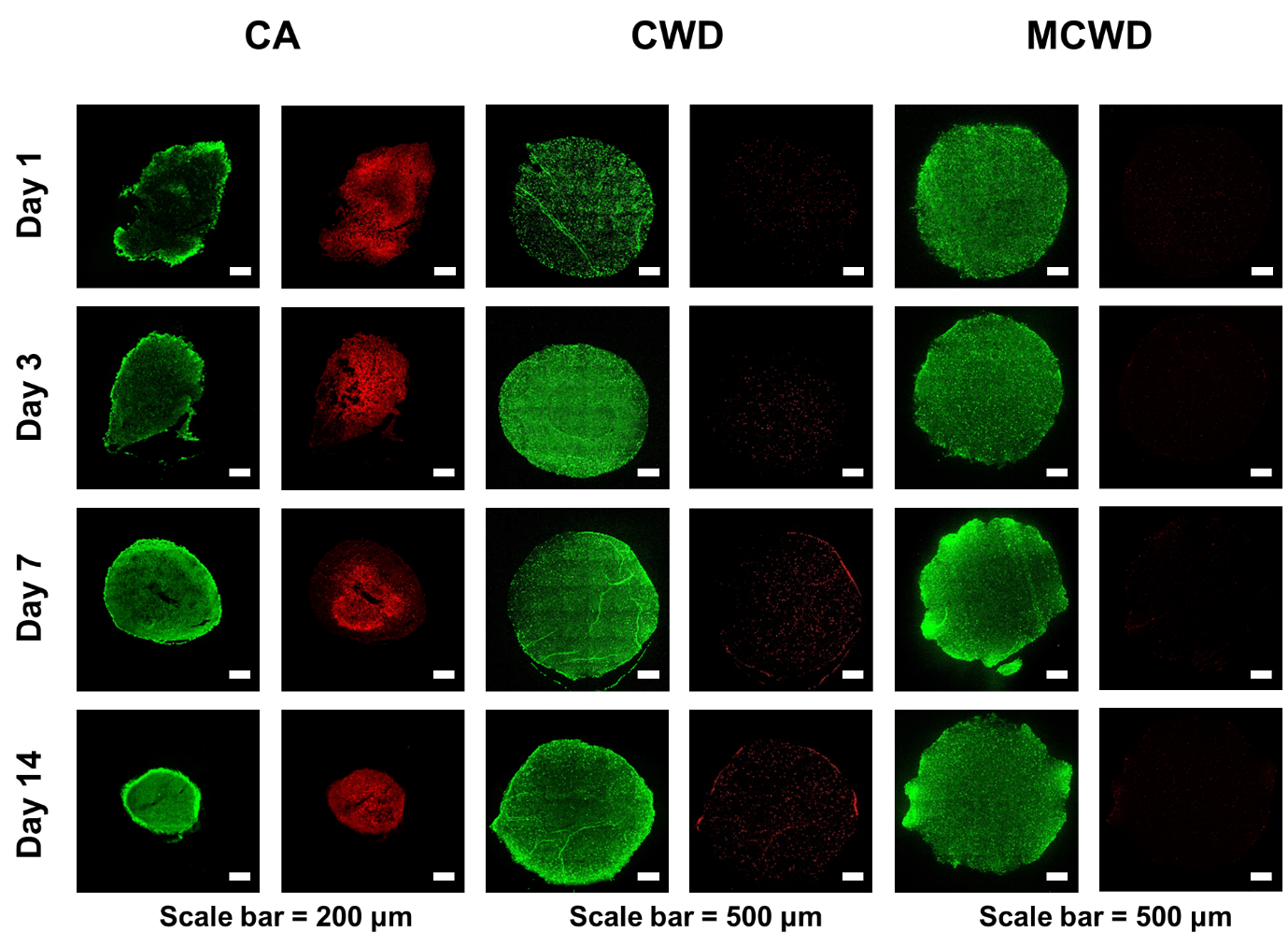


**Figure S5.** Live/Dead assay results showing cytotoxicity and cell viability of hADSCs over time in CA, CWD, and MCWD, highlighting survival rates at different time points


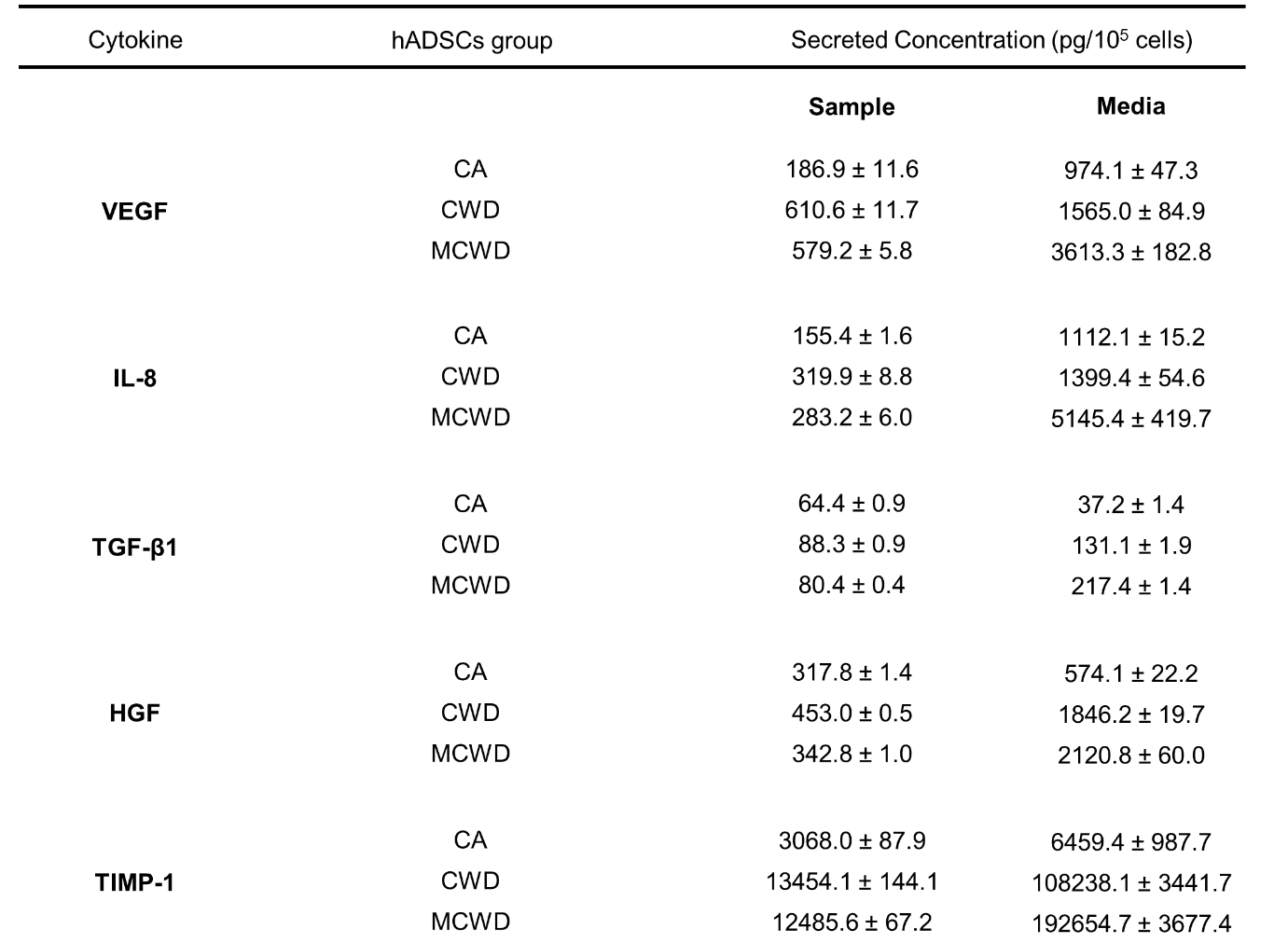


**Figure. S6.** ELISA analysis comparing the secretion efficiency of CA, CWD, and MCWD, both within the sample and into the media, to evaluate the release profiles of paracrine factors.


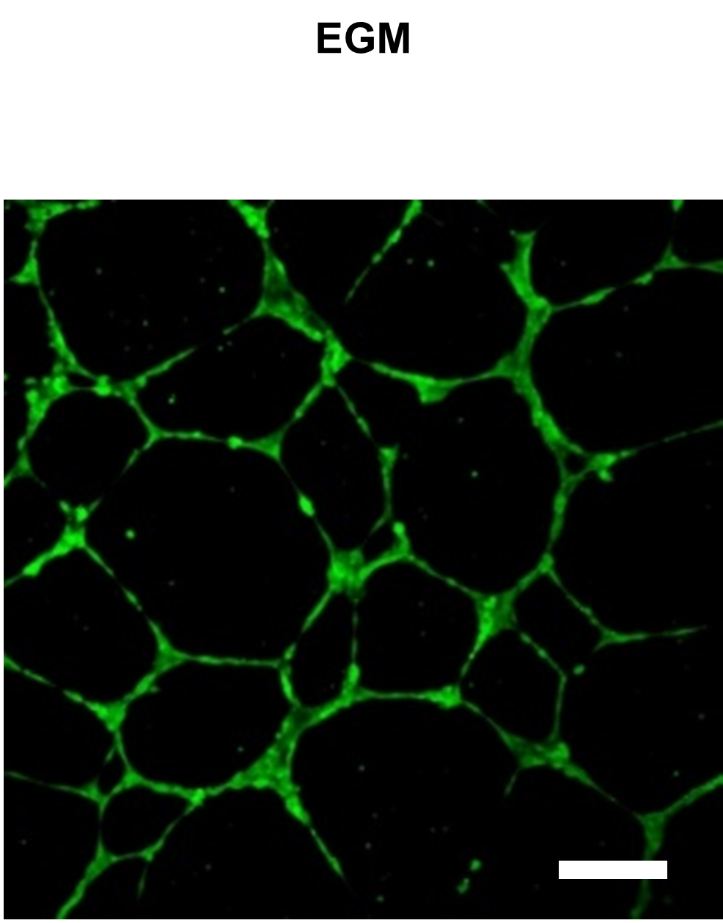


**Figure S7.** Confocal fluorescence images of HUVEC tube formation assay results using EGM media.

**
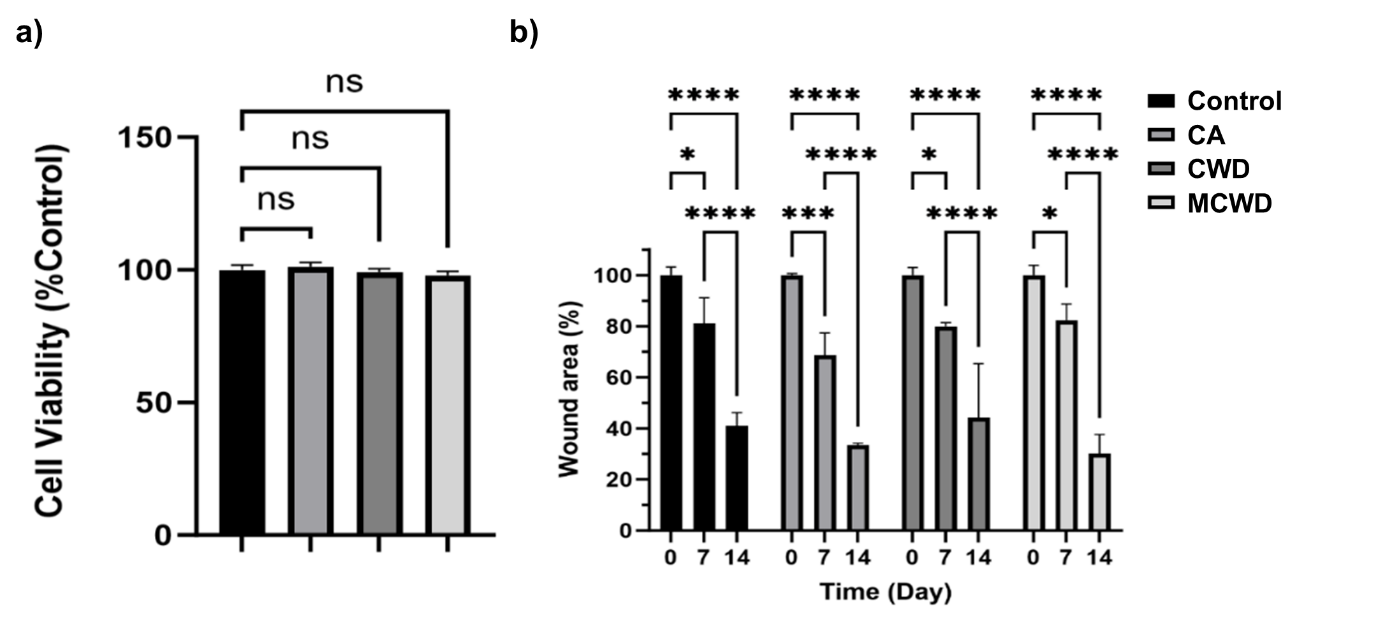
**

**Figure S8.** *In vivo* analysis demonstrating the therapeutic effects of CA, CWD, and MCWD compared to the control group. (a) CCK-8 analysis conducted prior to *in vivo* experiments to confirm the toxicity and biocompatibility profile of the treatments; (b) Evaluation of the wound contraction rate to assess the inhibitory effect on muscle-mediated wound contraction.

**
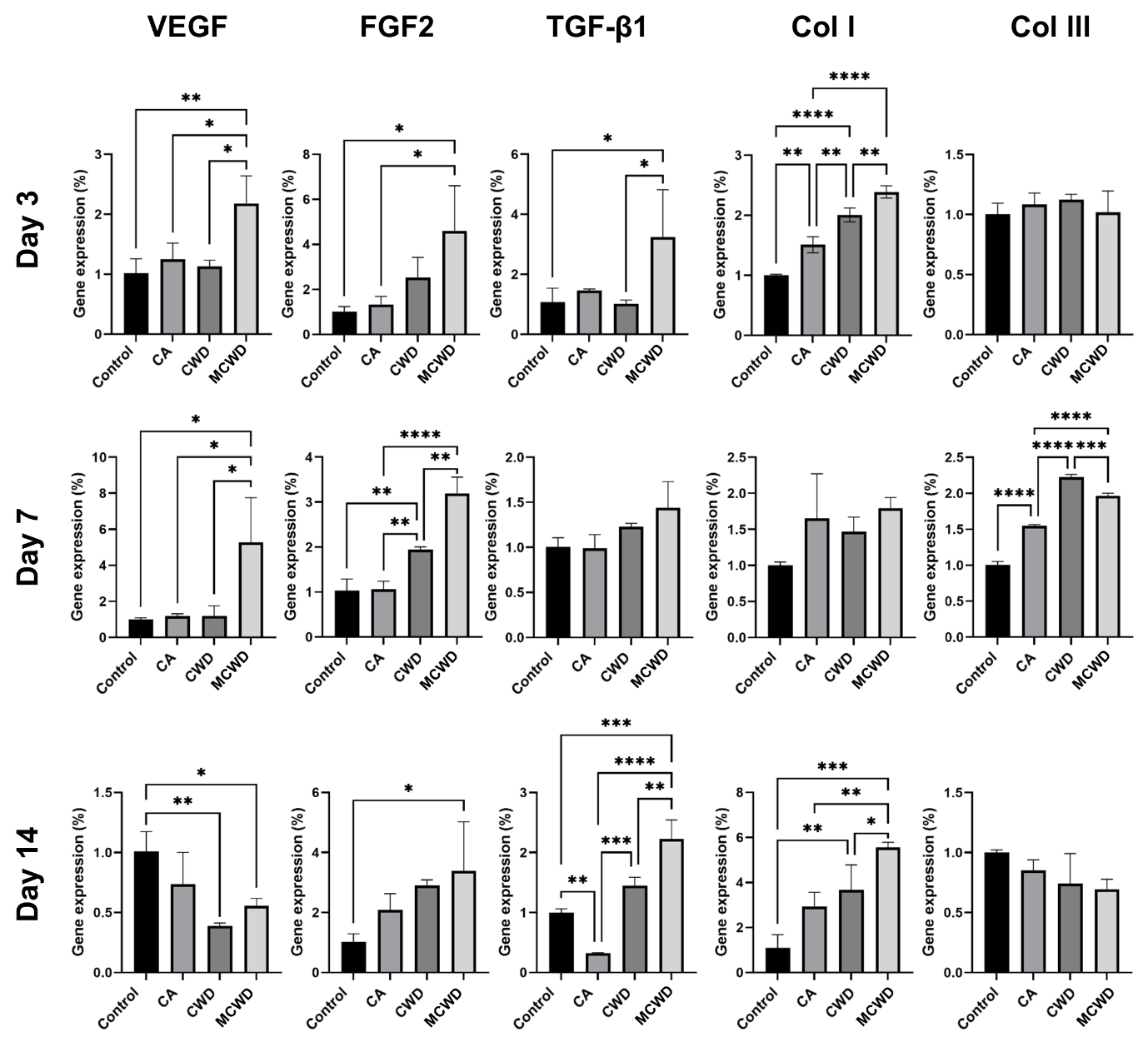
**

**Figure S9.** *In vivo* qPCR analysis of cytokine expression and collagen synthesis during wound healing process (n = 3).
